# Error-related brain state analysis using electroencephalography in conjunction with functional near-infrared spectroscopy during a complex surgical motor task

**DOI:** 10.1186/s40708-022-00179-z

**Published:** 2022-12-09

**Authors:** Pushpinder Walia, Yaoyu Fu, Jack Norfleet, Steven D. Schwaitzberg, Xavier Intes, Suvranu De, Lora Cavuoto, Anirban Dutta

**Affiliations:** 1 Neuroengineering and Informatics for Rehabilitation Laboratory, Department of Biomedical Engineering, University at Buffalo, Buffalo, USA; 2 Department of Industrial and Systems Engineering, University at Buffalo, Buffalo, USA; 3 U.S. Army Futures Command, Combat Capabilities Development Command Soldier Center STTC, Orlando, USA; 4 University at Buffalo School of Medicine and Biomedical Sciences, Buffalo, USA; 5 Center for Modeling, Simulation and Imaging in Medicine, Rensselaer Polytechnic Institute, Troy, NY USA; 6 Department of Biomedical Engineering, Rensselaer Polytechnic Institute, Troy, USA; 7 Neuroengineering and Informatics for Rehabilitation and Simulation-Based Learning, University of Lincoln, Lincoln, UK

**Keywords:** fNIRS, EEG, Microstate analysis, Skill training, Error based learning

## Abstract

Error-based learning is one of the basic skill acquisition mechanisms that can be modeled as a perception–action system and investigated based on brain–behavior analysis during skill training. Here, the error-related chain of mental processes is postulated to depend on the skill level leading to a difference in the contextual switching of the brain states on error commission. Therefore, the objective of this paper was to compare error-related brain states, measured with multi-modal portable brain imaging, between experts and novices during the Fundamentals of Laparoscopic Surgery (FLS) “suturing and intracorporeal knot-tying” task (FLS complex task)—the most difficult among the five psychomotor FLS tasks. The multi-modal portable brain imaging combined functional near-infrared spectroscopy (fNIRS) and electroencephalography (EEG) for brain–behavior analysis in thirteen right-handed novice medical students and nine expert surgeons. The brain state changes were defined by quasi-stable EEG scalp topography (called microstates) changes using 32-channel EEG data acquired at 250 Hz. Six microstate prototypes were identified from the combined EEG data from experts and novices during the FLS complex task that explained 77.14% of the global variance. Analysis of variance (ANOVA) found that the proportion of the total time spent in different microstates during the 10-s error epoch was significantly affected by the skill level (*p* < 0.01), the microstate type (*p* < 0.01), and the interaction between the skill level and the microstate type (*p* < 0.01). Brain activation based on the slower oxyhemoglobin (HbO) changes corresponding to the EEG band power (1–40 Hz) changes were found using the regularized temporally embedded Canonical Correlation Analysis of the simultaneously acquired fNIRS–EEG signals. The HbO signal from the overlying the left inferior frontal gyrus—opercular part, left superior frontal gyrus—medial orbital, left postcentral gyrus, left superior temporal gyrus, right superior frontal gyrus—medial orbital cortical areas showed significant (*p* < 0.05) difference between experts and novices in the 10-s error epoch. We conclude that the difference in the error-related chain of mental processes was the activation of cognitive top-down attention-related brain areas, including left dorsolateral prefrontal/frontal eye field and left frontopolar brain regions, along with a ‘focusing’ effect of global suppression of hemodynamic activation in the experts, while the novices had a widespread stimulus(error)-driven hemodynamic activation without the ‘focusing’ effect.

**Supplementary Information:**

The online version contains supplementary material available at 10.1186/s40708-022-00179-z.

## Introduction

**Fig. 1:**
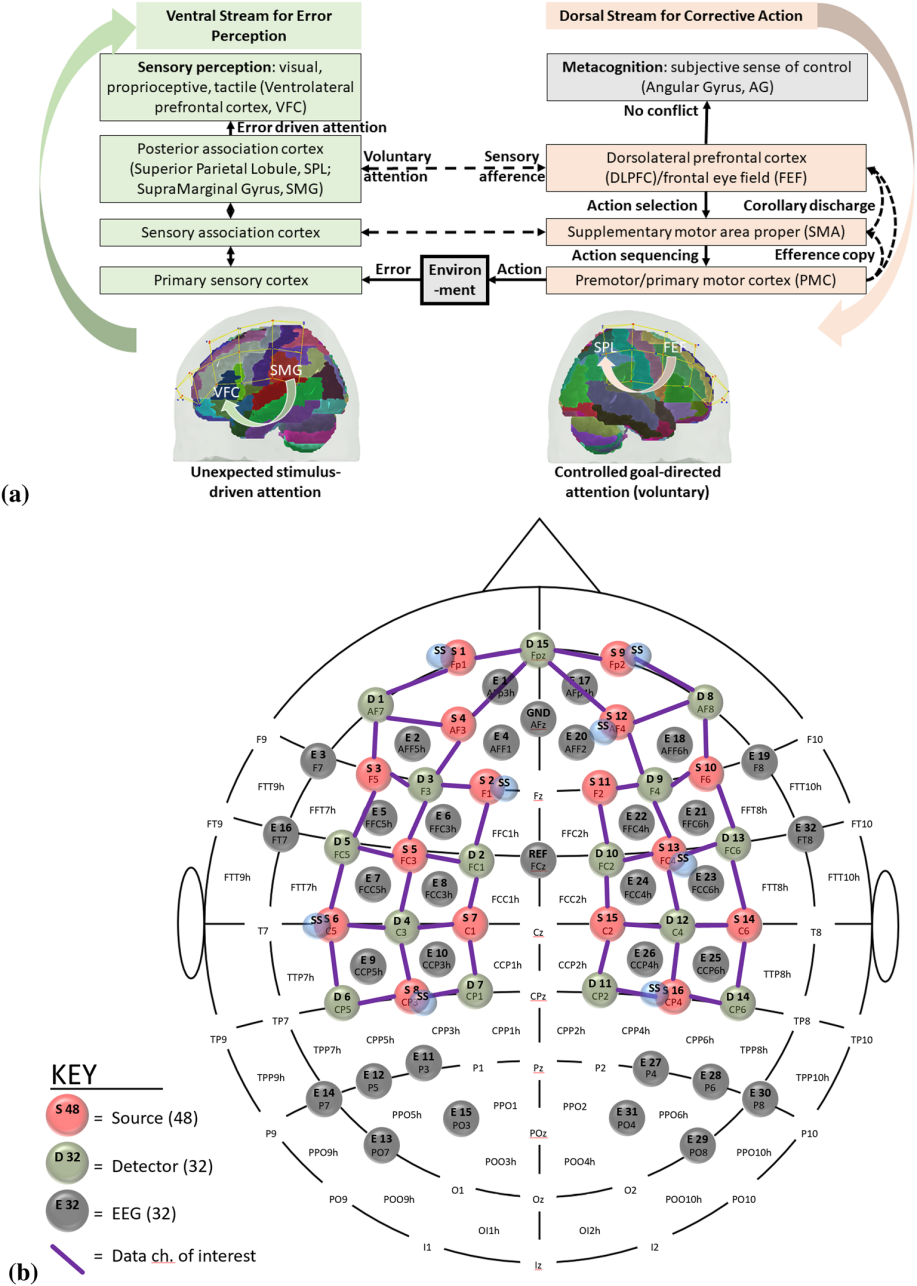

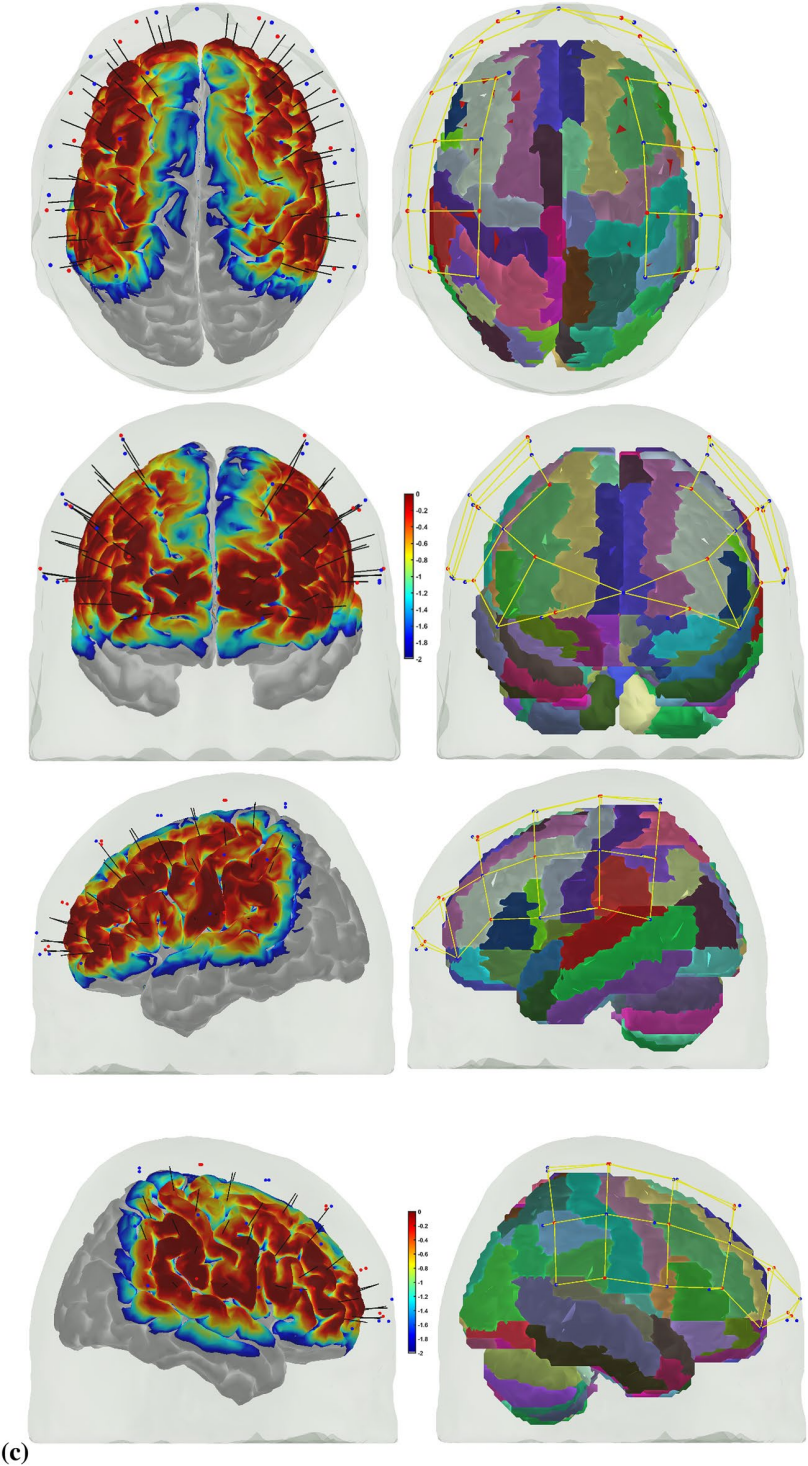

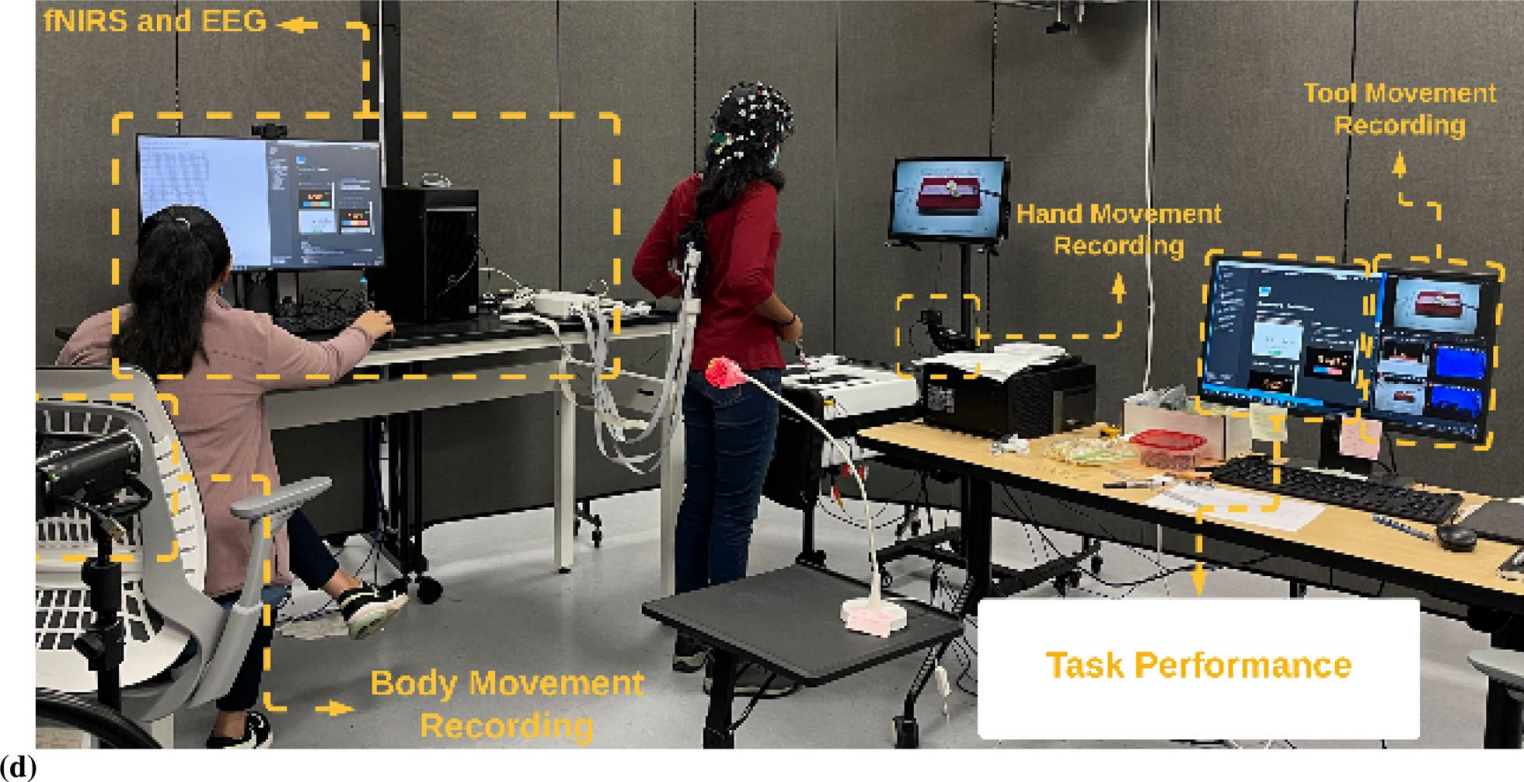
**a** Perception action system for error-related mental processes during laparoscopic surgical training. Portable neuroimaging allowed investigation of the brain regions based on the optode montage (see Fig. 1b) and its sensitivity profile (see Fig. 1c) that included ventrolateral prefrontal cortex (VFC), superior parietal lobule (SPL), supramarginal gyrus (SMG), angular gyrus (AG), dorsolateral prefrontal cortex (DLPFC), frontal eye field (FEF), premotor and primary motor cortex (PMC). A distinction is made between the unexpected stimulus (error) driven attention and the controlled goal directed attention in the frontoparietal network system. **b** Multi-modal (fNIRS–EEG) sensor montage including short-separation (labelled SS in light blue ellipses) channels. The red filled circles are the fNIRS sources, the green filled circles are the fNIRS detectors, the grey filled circles are the EEG electrodes, the violet lines are the optode pairs for the fNIRS channels. **c** Probe sensitivity values in the 0.01 to 1 range are displayed logarithmically as − 2 to 0 in log10 units in the color bar (left panels). The projection of the fNIRS channels on the cortex are shown with black arrows (left panels) along with the Automated Anatomical Labelling (AAL) of the brain regions in color (right panels). Table 1 shows the AAL of the cortical areas underlying fNIRS channels (source #–detector # pair). **d** Experimental setup in the laboratory with the subject performing the FLS "suturing and intracorporeal knot-tying" task (FLS complex task)

Error-based learning is one of the basic skill acquisition mechanisms involving error detection, error correction, and subsequent performance adjustments [85]. Here, individual differences in the error perception and attention reorientation for corrective action are postulated to differ between experts and novices. Notably, the error can be preemptively corrected by a predictive mechanism based on a forward model [103] that is postulated to improve with expertise. The brain can be considered an information processing system during skill acquisition. In that case, the investigation of the error-related states of the system in the experts and novices can provide insights into how the error event drives the attention reorientation for skilled corrective action. Notably, a distinction can be made between “internal monitoring” of error based on a predictive forward modeling framework [103] and “external monitoring” of error based on the action in the environment. In our prior work [46], we presented a perception–action model for brain–behavior analysis of laparoscopic surgical skill training. We showed the importance of the efference copy information from the motor cortices to the prefrontal cortex for postulated left-lateralized perceptual decision-making to reduce behavioral variability. Figure 1a shows the proposed perception–action link [46], where our optode montage (shown in Fig. 1b) captured the dorsal stream for action starting from action selection in the dorsolateral prefrontal cortex (PFC) to action sequencing in the supplementary motor area (SMA) to action performance in the primary motor cortex (PMC). Then, the efference copy information from the PMC is transmitted to the SMA and PFC, whereas the corollary discharge from the SMA is sent to the PFC. Then, any conflict (“internal monitoring” of error) with the sensory reafference is monitored by the angular gyrus for a subjective sense of agency [38]. The ventral stream for the perception of the sensory feedback (“external monitoring” of error) from the environment at the primary sensory cortex flows to the sensory association cortex and then to the posterior association cortex (e.g., supramarginal gyrus), leading to conscious error perception in the ventrolateral PFC (VLPFC). Here, the PFC interacts through reciprocal and reentrant connections with different areas of the posterior association cortex [27], including the superior parietal lobule (SPL) and supramarginal gyrus (SMG), to integrate the information from multiple sensory inputs and motor actions [54] for action perception [53]. These multiple visual streams are increasingly being established in humans via functional connectivity and diffusion tractography [81].

We postulate that the frontoparietal interactions can be divided into controlled goal-directed attention from the frontal eye field (FEF) to the SPL and unexpected stimulus(error)-driven attention from SPG to the VLPFC [17], which is crucial for error-based learning. Figure 1c shows the sensitivity profile of our optode montage, where automated anatomical labeling (AAL) [82] of the brain regions with Montreal Neurological Institute (MNI) coordinates is presented in Table 1. Here, we postulate that the left-lateralized perceptual decision-making is crucial for ‘bottom-up’ error perception. Then, cognitive, ‘top-down’ attention reorientation and right dorsolateral ‘top-down’ cognitive monitoring [91], e.g., in experts, when compared to ‘bottom-up’ control of attention reorientation, e.g., in novices, is proposed to be subserved by the dorsal posterior parietal and frontal regions of the brain [14] during the laparoscopic surgical task (Fig. 1d).

**Table 1:** Automated anatomical labeling (AAL) and Montreal Neurological Institute (MNI) coordinates of the cortical areas underlying fNIRS channels (source #–detector # pair) when projected on the cortex in AtlasViewer using its default head model [1]

Source #	Detector #	Channel coordinates (MNI)	AAL label names	Regions
1	1	− 30 56–7	Frontal_Inf_Orb_L	Inferior frontal gyrus, orbital part left hemisphere
1	15	− 9 64–8	Frontal_Sup_Orb_L	Superior frontal gyrus, orbital part left hemisphere
1	16	− 11 57–5	Frontal_Sup_Orb_L	Superior frontal gyrus, orbital part left hemisphere
2	2	− 17 26 48	Frontal_Sup_L	Superior frontal gyrus, dorsolateral left hemisphere
2	3	− 21 34 28	Frontal_Mid_L	Middle frontal gyrus left hemisphere
2	17	− 13 45 38	Frontal_Sup_L	Superior frontal gyrus, dorsolateral left hemisphere
3	1	− 35 45 to 2	Frontal_Inf_Orb_L	Inferior frontal gyrus, orbital part left hemisphere
3	3	− 30 33 20	Frontal_Mid_L	Middle frontal gyrus left hemisphere
3	5	− 36 8 13	Frontal_Inf_Oper_L	Inferior frontal gyrus, opercular part left hemisphere
4	1	− 38 55 to 2	Frontal_Mid_Orb_L	Superior frontal gyrus, medial orbital left hemisphere
4	3	− 37 48 14	Frontal_Inf_Tri_L	Inferior frontal gyrus, triangular part left hemisphere
4	15	− 16 61 0	Frontal_Sup_Orb_L	Superior frontal gyrus, orbital part left hemisphere
5	2	− 47 12 53	Frontal_Mid_L	Middle frontal gyrus left hemisphere
5	3	− 47 24 31	Frontal_Mid_L	Middle frontal gyrus left hemisphere
5	4	− 47 to 9 41	Postcentral_L	Postcentral gyrus left hemisphere
5	5	− 58 11 28	Precentral_L	Precental gyrus left hemisphere
6	4	− 46 to 23 34	Postcentral_L	Postcentral gyrus left hemisphere
6	5	− 57 to 11 15	Temporal_Sup_L	Superior temporal gyrus left hemisphere
6	6	− 37 to 34 16	Rolandic_Oper_L	Rolandic operculum left hemisphere
6	18	− 63 to 19 18	SupraMarginal_L	Supramarginal gyrus left hemisphere
7	2	− 27 to 4 63	Frontal_Sup_L	Superior frontal gyrus, dorsolateral left hemisphere
7	4	− 50 to 22 63	Postcentral_L	Postcentral gyrus left hemisphere
7	7	− 18 to 34 57	Postcentral_L	Postcentral gyrus left hemisphere
8	4	− 46 to 30 47	Postcentral_L	Postcentral gyrus left hemisphere
8	6	− 52 to 49 34	SupraMarginal_L	Supramarginal gyrus left hemisphere
8	7	− 32 to 47 54	Parietal_Inf_L	Inferior parietal, but supramarginal and angular gyri left hemisphere
8	19	− 29 to 43 40	Parietal_Inf_L	Inferior parietal, but supramarginal and angular gyri left hemisphere
9	8	41 57 to 8	Frontal_Mid_Orb_R	Middle frontal gyrus, orbital part Right hemisphere
9	15	15 67 to 11	Frontal_Sup_Orb_R	Superior frontal gyrus, orbital part right hemisphere
9	20	21 54 to 1	Frontal_Sup_R	Superior frontal gyrus, dorsolateral right hemisphere
10	8	47 47 to 2	Frontal_Inf_Orb_R	Inferior frontal gyrus, orbital part right hemisphere
10	9	51 37 18	Frontal_Inf_Tri_R	Inferior frontal gyrus, triangular part right hemisphere
10	13	55 14 10	Frontal_Inf_Oper_R	Inferior frontal gyrus, opercular part right hemisphere
10	21	44 19 8	Frontal_Inf_Tri_R	Inferior frontal gyrus, triangular part right hemisphere
11	9	23 36 32	Frontal_Sup_R	Superior frontal gyrus, dorsolateral right hemisphere
11	10	33 34 56	Frontal_Sup_R	Superior frontal gyrus, dorsolateral Right hemisphere
12	8	42 53 to 1	Frontal_Mid_Orb_R	Middle frontal gyrus, orbital part right hemisphere
12	9	49 56 16	Frontal_Mid_R	Middle frontal gyrus Right hemisphere
12	15	18 62 to 1	Frontal_Sup_Orb_R	Superior frontal gyrus, orbital part right hemisphere
13	9	53 24 32	Frontal_Inf_Tri_R	Inferior frontal gyrus, triangular part right hemisphere
13	10	45 9 47	Precentral_R	Precental gyrus right hemisphere
13	12	57 to 7 46	Precentral_R	Precental gyrus right hemisphere
13	13	58 9 26	Precentral_R	Precental gyrus right hemisphere
13	22	48 6 38	Precentral_R	Precental gyrus Right hemisphere
14	12	63 to 20 36	SupraMarginal_R	Supramarginal gyrus Right hemisphere
14	13	43 to 8 18	Insula_R	Insula right hemisphere
14	14	46 to 35 18	Temporal_Sup_R	Superior temporal gyrus right hemisphere
15	10	36 to 7 64	Frontal_Sup_R	Superior frontal gyrus, dorsolateral right hemisphere
15	11	39 to 38 76	Postcentral_R	Postcentral gyrus right hemisphere
15	12	41 to 22 52	Precentral_R	Precental gyrus right hemisphere
16	11	42 to 49 57	Parietal_Sup_R	Superior parietal gyrus right hemisphere
16	12	51 to 33 49	SupraMarginal_R	Supramarginal gyrus right hemisphere
16	14	45 to 46 34	Angular_R	Angular gyrus Right hemisphere
16	23	35 to 46 42	Angular_R	Angular gyrus right hemisphere

In the current study, we followed the Fundamentals of Laparoscopic Surgery (FLS) which is a common education and training module designed for medical residents, fellows, and physicians to provide them with a set of basic surgical skills necessary to conduct laparoscopic surgery successfully. The FLS training is a joint education program between the Society of American Gastrointestinal Endoscopic Surgeons and the American College of Surgeon to establish box trainers (physical simulators) in standard surgical training curricula [8]. It was introduced to systemize training and evaluation of cognitive and psychomotor skills required to perform minimally invasive surgery. FLS certification in general surgery in the USA uses five psychomotor tasks with increasing task complexity: (i) pegboard transfers, (ii) pattern cutting, (iii) placement of a ligating loop, (iv) suturing with extracorporeal knot tying, and (v) suturing with intracorporal knot tying. Therefore, understanding the brain–behavior relationship during error-based learning is necessary for informed training and assessment [19]. In the current study, we investigated the FLS “suturing and intracorporeal knot-tying” task, which is the most difficult among the five psychomotor tasks that surgeons must pass as part of the board certification process. This skill enables surgeons to provide a wide range of advanced surgical procedures [3], however, acquiring this skill needs protracted training. The skilled behavior can be characterized as a coordinated spatio-temporal 3D movement using 2D camera feedback with the interaction between the body and the environment within a restricted surgical volume. FLS “suturing and intracorporeal knot-tying” is a complex motor task requiring high precision hand–eye coordination, depth perception in the 2D view and tool control for optimal performance [39]. Here, an investigation of the brain state changes following an error event during perturbations in the performance, i.e., one of the basic principles of motor skill acquisition [20], may provide insights into the error-related brain–behavior relationship in experts and novices.

The error-related brain–behavior relationship can be investigated using an integrated approach to perception and action [34] that we presented in our prior work [46]. Relying on the sensory error feedback (“external monitoring” of error) does not allow preemptive error correction that is expected in skilled behavior so a forward (internal) model is expected to make sensory error predictions (“internal monitoring” of error) that can be used to continually update forthcoming motor commands [85] for error correction. In addition, the adaptive internal model of the body and the environment is continuously learned from sensory prediction errors (Shadmehr et al., 2010) to perform goal-directed action ‘expertly’ using noisy and delayed sensory feedback. However, error correction requires reorientation from ongoing goal-directed attention subserved by intraparietal sulcus (IPS)/superior parietal lobule (SPL) and frontal eye field (FEF) to error-stimulus-driven attention subserved by inferior frontal gyrus (IFG)/middle frontal gyrus (MFG) and temporoparietal junction (TPJ), where switching may have an implicit cost for the brain [84]. Therefore, brain state changes following the error event can provide insights into attention reorientation necessary for deliberate practice [23] despite the cost. Indeed, an increased speed of action selection at the expense of cognitive flexibility [74, 94] to adapt the internal model can lead to automaticity despite the residual error that will be detrimental to laparoscopic surgery training.

In the current study, we postulate that the error-related brain response will involve contextual switching of the brain state necessary for error perception and corrective action [7]. We further postulate that this contextual switching of the brain state can be captured by microstates [64] that are global patterns of quasi-stable (60–120 ms) scalp potential topographies of the large-scale brain networks [59]. For example, brain response related to post-error slowing vis-à-vis perceptual processing and post-error corrective action [70] can be considered contextual switching on error commission, where the scalp topographies have been found to reflect the role of the prefrontal cortex in error perception and the role of the premotor areas in the post-error adjustments [70]. Here, we postulate that the subjective error awareness or perception is critical [102], i.e., in the absence of error perception (“external monitoring” of error at the VLPFC, see Fig. 1a), the perception–action cycle for post-error adjustments will be missing [32] in those subjects. In addition, anterior cingulate/medial frontal cortex associated “internal monitoring” of error, e.g., error-related negativity, is postulated to be crucial for motor skill learning, where anterior cingulate/medial frontal cortex activity is known to scale with motor error [85]. The scalp potential topography for error-related negativity signal has a prominent fronto-central radial voltage distribution [102] that is postulated to be generated due to the negative reinforcement signal to the anterior cingulate cortex via the mesencephalic dopamine system [41]. Then, the negative reinforcement signal at the anterior cingulate cortex (“internal monitoring”) and/or the left-lateralized error perceptual (“external monitoring”) decision-making can trigger right-lateralized executive control of attention [17] that activates the premotor areas for post-error adjustments [41, 70]. Here, post-error adjustments in experts are postulated to be preemptive (based on “internal monitoring”) subthalamic nucleus (STN)-mediated hyper direct stopping with global suppressive effects [29] followed by the activation of motor semantics [77] accompanied by the implicit activation of corrective motor representations (van Elk et al. 2009).

We evaluated a portable brain–behavior approach based on functional near-infrared spectroscopy (fNIRS) in conjunction with electroencephalogram (EEG) [49] during FLS skill training using the FLS Trainer Box device [24] to capture brain responses subserving error processing during the FLS “suturing and intracorporeal knot-tying” task (henceforth, the FLS complex task). Here, the change in the EEG scalp topography during error processing after error commission is analyzed as a sequence of "microstate" during which the scalp potential field remains semi-stable [64]. Microstate analysis leverages the excellent temporal resolution of EEG [64] and a meta-criterion on global field power [89], favoring the highest signal-to-noise ratio [16]. The proposed computational circuit mechanisms [37] have presented selective attention [15] as cortical excitability alterations by the thalamus [43] acting as a “spotlight,” which is postulated for the error-related brain state changes [44]. Here, the microstate approach for a brain state correlates of the response [73] to error has a crucial a priori assumption that only one spatial topography map entirely defines the relevant global state of the brain at each moment in time and the residuals are considered noise.

Microstate analysis has been validated based on resting-state functional magnetic resonance imaging (fMRI), showing a close relationship of the microstates with resting-state brain networks [64]. Since fMRI is challenging [55, 101], during the FLS complex task, therefore, we combined EEG with fNIRS—a non-invasive optical imaging technique [95] that exploits neurovascular coupling (like fMRI) to measure cortical activity. Combining fNIRS with EEG is beneficial, since EEG can measure neuronal activity at a high temporal resolution for microstate analysis. In contrast, fNIRS can uncover cortical correlates of microstates under the neurovascular coupling phenomenon [49, 88, 90]. Microstate prototypes were selected from the excellent temporal resolution of EEG [64] and the meta-criterion for global field power (GFP) [16]. Then, the EEG band power changes corresponding to the oxyhemoglobin (HbO) concentration changes from fNIRS data were found using regularized temporally embedded Canonical Correlation Analysis (tCCA). This allowed analysis of the cortical activation based on HbO changes at the brain regions associated with the localized EEG scalp “hot spots” in the experts and novices. While EEG detected fast changes under the limitations of volume conduction (addressed with surface Laplacian [48]), fNIRS provided a corresponding hemodynamic response over a longer timeframe with better localization due to its limited spatial sensitivity. Here, human error processing [41] is proposed to be different in experts and novices due to their differences in the error-related mental processes measured in this study with simultaneously acquired EEG and fNIRS signals. Given each modality's different characteristics and physiological information, simultaneously acquired EEG and fNIRS signals are postulated to provide mechanistic insights into the brain state changes during error processing.

## Materials and methods

### Subjects and task

**Fig. 2:**
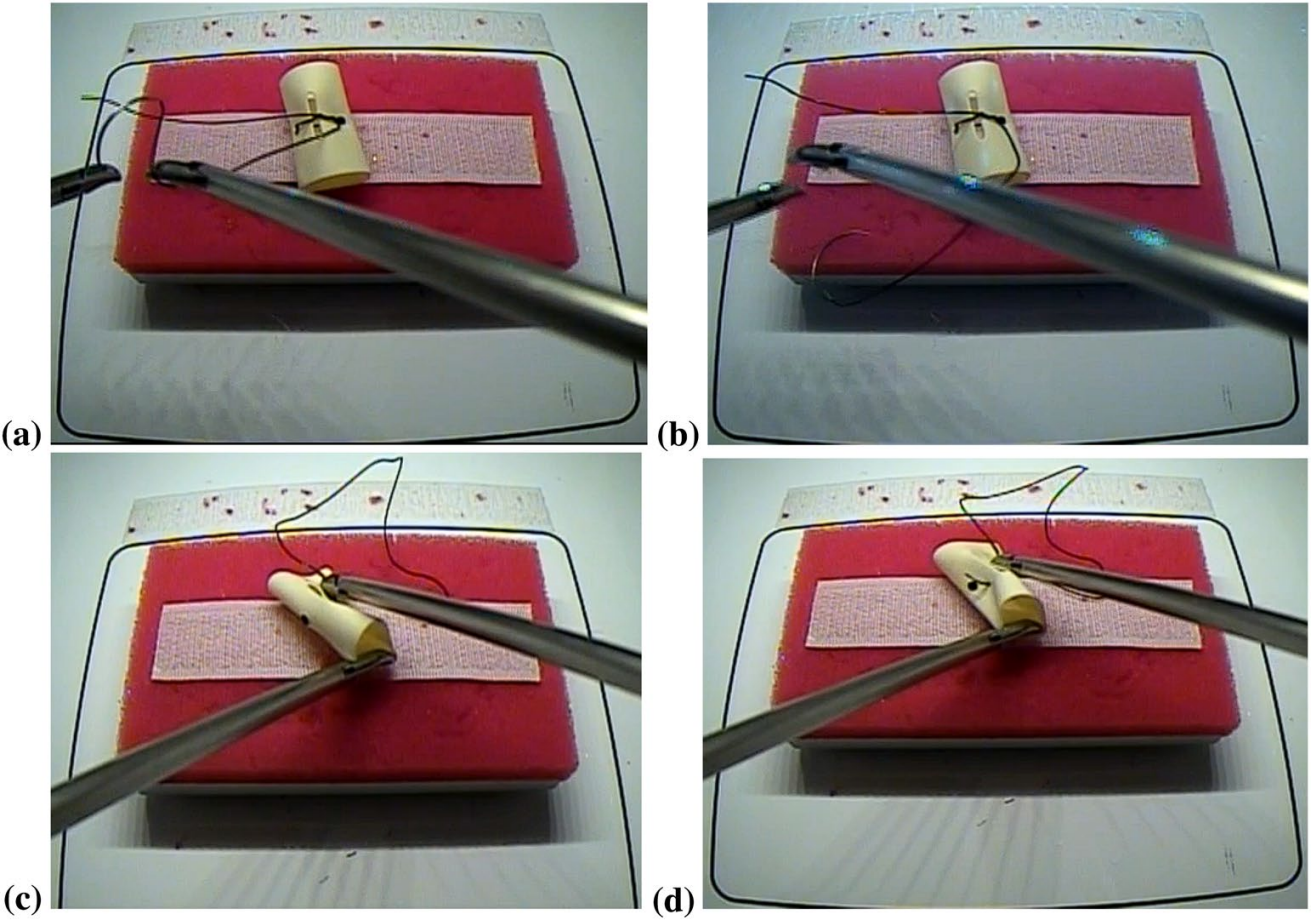
**a**, **b** Image sequence showing “needle drop” error event from (**a**) to (**b**) during task performance. **C,** **d** Image sequence showing “incorrect needle insertion” error event from (**c**) to (**d**) during task performance

Thirteen right-handed healthy novice medical students and nine right-handed expert surgeons were recruited after written consent for the study. The study was approved by the Institutional Review Board of the University at Buffalo, USA. All study procedures were performed according to the local human subjects' research regulations. The experts (attending surgeons and residents) had greater than 1-year experience with laparoscopic tasks, whereas the novices (medical students) had never experienced the laparoscopic task. All the subjects were instructed verbally with a standard set of instructions on completing the FLS “suturing and intracorporeal knot-tying” task to the best of their capacity. Participants were provided with two laparoscopic needle drivers, one suturing scissors, and a needle with a suture of 15 cm in length. In this FLS complex task, a Penrose drain with marked targets is placed on the Velcro strip inside the FLS Trainer Box. The subject has to tie three throws of a knot intracorporeally using two needle drivers, where the last two knots are single throws followed by a double throw, which closes the slit in the Penrose drain [79]. The task involves inserting the suture through two marks in a Penrose drain and then tying a double-throw knot followed by two single-throw knots using two needle graspers operated by both hands. The FLS complex task starts when the subject picks up the suture and the needle driver on the ‘start’ command and ends when the subject cuts both ends of the suture, where the task completion is limited to 10 min (600 s). The task was repeated three times along with 2 min of the rest period, and the 'start' and 'stop' triggers for the FLS task were manually registered with the data acquisition software. The experimenter labeled using the FLS box camera view of the error events at the “needle drop” and “incorrect needle insertion,” as shown in Fig. 2a–d, respectively. The multimodal imaging system using simultaneously acquired EEG and fNIRS signals recorded concurrent electrophysiological and hemodynamic brain responses, while the subject performed the FLS complex task that included error events.

### Synchronized multimodal portable brain imaging

A customized montage consisting of EEG electrodes and fNIRS optodes was used to record synchronized multimodal brain activation signals. 32-channel EEG signals were recorded using a wireless LiveAmp system (Brain Vision, USA). EEG recordings were obtained at 500 Hz using active gel-electrodes. 32-channel fNIRS signals and 8-channel short-separation channels were recorded at a 5 Hz sampling rate with NIRSPORT2 (NIRx, USA). A 1 Hz hardware trigger signal implemented the fNIRS–EEG synchronization, and the multimodal data were aligned and epoched in 1-s time windows. The optical probes and electrodes were located following standard 10–5 montage (see Fig. 1b), with fNIRS probe sensitivity [1] shown in Fig. 1c. The probes were carefully placed on the subject's head to avoid hair interference and to not hinder the subject's mobility during the mobile brain–behavior study (see Fig. 1d). Table 1 (from AtlasViewer software using its default head model) lists the labels of the fNIRS cortical region of interest (ROIs) that are based on the Automated Anatomical Labelling atlas [82], 3) and Montreal Neurological Institute coordinate space [1].

### fNIRS–EEG data preprocessing

The simultaneously recorded EEG and fNIRS signals were preprocessed and analyzed offline. The EEG signals were preprocessed using the open-source EEGlab toolbox (https://sccn.ucsd.edu/eeglab/index.php) for the microstate analysis [64]. Specifically, the data were downsampled to 250 Hz and high-pass filtered at 1 Hz. Then, the line noise was removed using the 'cleanline' function, followed by the ‘clean_rawdata’ function to reject bad channels. The bad channels were interpolated using spherical splines [71] in ‘clean_rawdata’ function followed by re-referencing to the global average. Artifact subspace reconstruction (ASR) was performed using the default settings in EEGlab, followed by re-referencing to the global average. ASR is an automated method based on a user-specified parameter that can effectively remove transient EEG artifacts [11]. We used the default ASR parameter value of 20, while the optimal value is between 20 and 30 to balance between removing non-brain signals and retaining brain activities [11].

The preprocessed EEG data from 13 novices and eight experts were used for the microstate analysis, since we rejected one expert subject to keep the maximum number of bad channels for any subject less than five. Then, the Laplacian spatial filter was applied to remove the volume conduction from the subcortical sources while keeping the cortical sources that corresponded with the hemodynamic response measured with fNIRS. The fNIRS data were processed using the standard open-source HOMER3 package (https://github.com/BUNPC/Homer3). The fNIRS preprocessing pipeline consists of the following: first, the intensity was converted to optical density, and then motion artifacts were detected and filtered with the help of the Savitzky–Golay filtering method [45] with default parameters in HOMER3. Then, the optical density was bandpass filtered in the neurovascular coupling band, 0.01–0.1 Hz, and then converted to chromophore (HbO) concentration with unit partial pathlength factor.

### Error-related fNIRS–EEG microstates analysis

Microstate analysis was performed using the EEGlab toolbox [76] after aggregating EEG data during the FLS complex task from all the experts and novices. First, we identified EEG microstate prototypes based on modified K-means clustering available in the EEGlab toolbox. The candidate EEG prototypes are extracted with a high signal-to-noise ratio from the peaks of the global field power (GFP)[64]:

$$GFP=\sqrt{\left(\frac{1}{N}\sum_{i=1}^{N}{\left({EEG}_{i}(t)-\stackrel{-}{EEG(t)}\right)}^{2}\right)}$$

where ${EEG}_{i}\left(t\right)$ refers to the EEG signal at ith electrode and timepoint 't', and $\stackrel{-}{EEG(t)}$ is the average EEG signal across all the electrodes at the time 't'. The EEG microstate prototypes are then found from topographical clustering, where the scalp topography within clusters has the highest spatial similarity. Hierarchical clustering (such as Atomize and Agglomerate Hierarchical Clustering and Topographic Atomize and Agglomerate Hierarchical Clustering) has been shown to deliver similar performance to the K-means clustering [97]. In this study, the modified K-means clustering was used based on the goodness of fit of the microstate segmentation determined from the global explained variance (GEV) and the cross-validation (CV) criterion to select an appropriate number of clusters or microstates:

$$GEV=\frac{\sum_{t=1}^{L}{(Corr\left(EEG\left(t\right),{EEG}_{m}\left(t\right)\right)*GFP(t))}^{2}}{\sum_{t=1}^{L}{GFP(t)}^{2}}$$

where $EEG\left(t\right)$ is the EEG topography map at the time 't', ${EEG}_{m}\left(t\right)$ is the assigned microstate at the time 't', $Corr()$ is the spatial similarity between the two topography maps, and the L is the total number of timepoints for the analysis:

$$CV={\sigma }^{2}{ \left(\frac{C-1}{C-K-1}\right)}^{2}$$

where ${\sigma }^{2}$ is the estimator of the variance in the residual noise, C is the number of EEG channels, and K is the number of clusters or microstates [76].

Here, the GEV criterion should theoretically become monotonically larger with the increasing number of clusters, while the CV criterion should reach a minimum for an appropriate number of clusters or microstates [76]. In this study, the modified K-means clustering in the EEGlab toolbox found topographical maps of polarity invariant microstate prototypes [76] from the spontaneous EEG data acquired during the FLS complex task (including rest periods in between the trials). The GFP peaks were used to segment the spontaneous EEG time series with the minimum peak distance set at 10 ms (default), and 1000 randomly selected peaks (default) per subject were used for the segmentation. Then, we rejected the GFP peaks that exceeded one time the standard deviation of all the GFPs of all maps and segmented the EEG data into a predefined number (2 to 8) of microstates. Here, the goal is to maximize the similarity between the EEG samples and the prototypes of the microstates they are assigned to using the modified K-means algorithm [76]. The modified K-means algorithm also sorts the microstate prototypes in decreasing GEV. We had set 100 random initializations and 1000 maximum number of iterations for the modified K-means algorithm with the 1e-6 (default) as the relative threshold of convergence [76]. These microstates provided the prototypes for the subsequent FLS complex task-related and error-related microstate analysis.

Microstate labels were applied to the EEG samples based on topographical similarity, called 'backfitting', using the EEGlab toolbox to quantify the dynamic brain states during the start of the FLS complex task and the error epochs. The topographical similarity was found using the global map dissimilarity (GMD), which is a distance measure that is invariant to the strength of the EEG signal [76]. GMD measure quantifies how similar the topographical maps look:

$$GMD=\frac{\Vert \frac{EEG\left(t\right)}{GFP(t)}-\frac{{EEG}_{m}\left(t\right)}{{GFP(t)}_{m}}\Vert }{\sqrt{C}}$$

where $EEG\left(t\right)$ is the EEG topography map at the time ‘t’, ${EEG}_{m}\left(t\right)$ is the candidate microstate for backfitting at the time ‘t’, and C is the number of EEG channels.

The error epochs were defined for the 10-s duration following the error commission at the needle drop or incorrect needle insertion. Notably, a long enough duration of 10 s for the error epoch was chosen for the error evoked fNIRS–EEG response to capture the hemodynamic response function corresponding to the EEG band power (1–40 Hz) changes, since the maximum fNIRS frequency is 0.1 Hz in the neurovascular coupling band (i.e., a time period of 10 s). In addition, prior work [60] showed that the HbO concentration peaked in the time range of 3–9 s for complex motor action, so a 10-s duration was considered adequate for the hemodynamic response function in the error epoch as well during the 10 s at the start of the FLS complex task. The statistical properties of the EEG microstates were computed following temporal smoothing, since short periods of unstable EEG topographies can occur. The statistical properties of the EEG microstates were used to compare error-related cortical activation between the experts and the novices, e.g., average GFP, average GEV, average spatial Correlation, as well as the temporal properties, Occurrence, i.e., the average number of times per second a microstate is dominant, the Duration, i.e., the average duration of a given microstate (in milliseconds), and the Coverage, i.e., the fraction of time a given microstate is active.

**Fig. 3:**
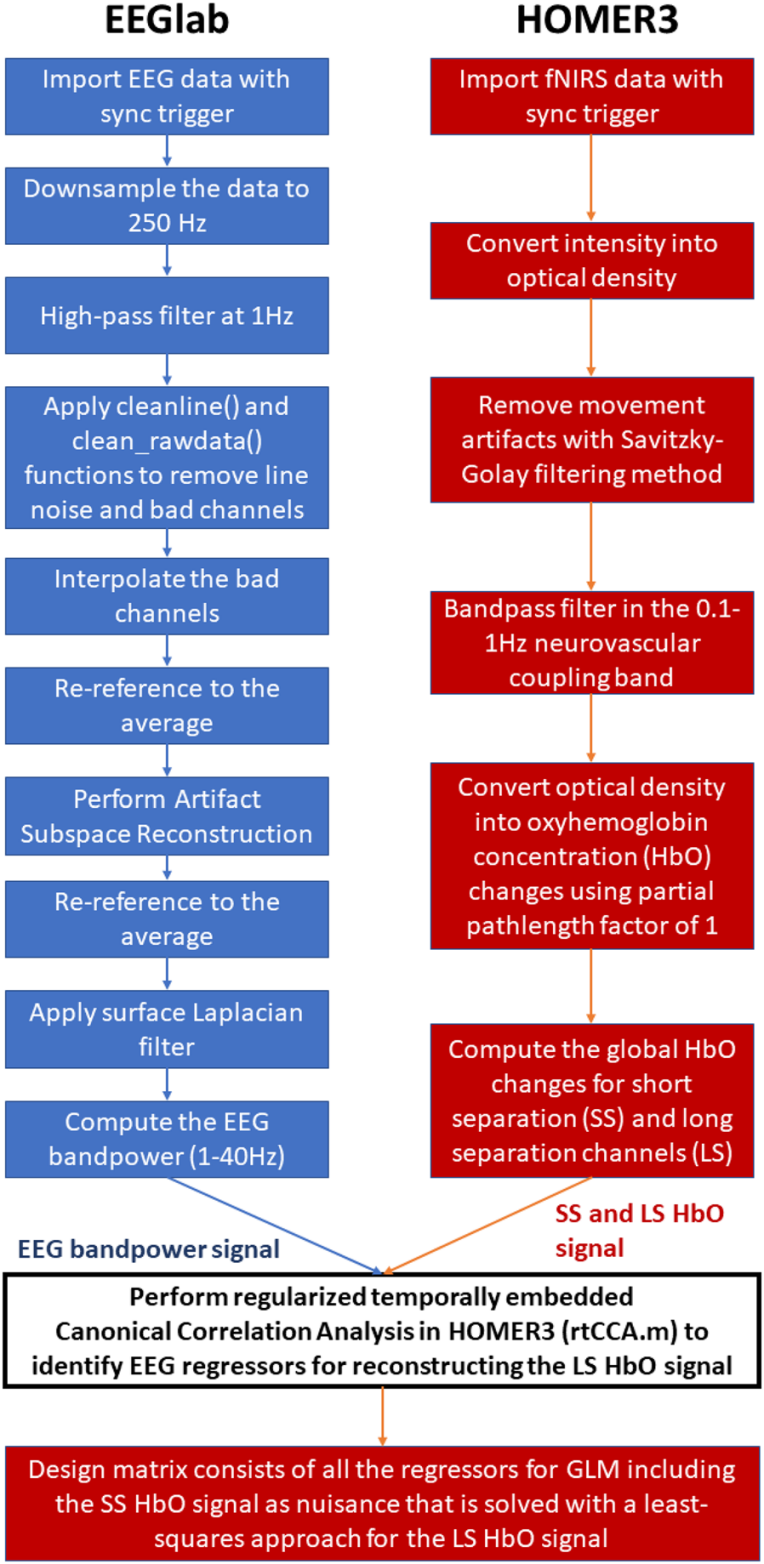
Flowchart of the steps for fNIRS–EEG preprocessing in HOMER3 and EEGlab, respectively, and the fNIRS post-processing using the EEG regressors and the short-separation HbO regressors in HOMER3

The correspondence between the fNIRS HbO changes and the EEG band power (1–40 Hz) changes was found based on the General Linear Model (GLM) and regularized Canonical Correlation Analysis with temporal embedding in HOMER3 [96]. The evoked hemodynamic signal is typically reconstructed with a weighted set of temporal basis functions in HOMER3 [96]; however, we reconstructed the HbO response from multi-channel EEG band power (1–40 Hz signals. Here, the design matrix consisted of all the regressors for GLM that are solved with a least-squares approach for each regressor's contribution based on their coefficients [96]. The GLM approach also captures systemic artefacts with short-separation (SS) fNIRS channels as regressors and a 3rd order polynomials to model drift. Therefore, the SS fNIRS channels served as the nuisance regressors for the systemic artefact in the design matrix [96], and the coefficients of the EEG band power (1–40 Hz) regressors were used to reconstruct the corresponding hemodynamic signal (HbO time series). Identification of the EEG band power (1–40 Hz) regressors from multi-channel EEG data was performed using the ‘hmrR_tCCA’ function in HOMER3 to find the neurovascular coupling in the latent space [78] between the HbO time series at all the long-separation (LS) fNIRS channels and the simultaneously acquired EEG band power (1–40 Hz) signals from all the EEG electrodes. Here, we selected 15 regressors from simultaneously acquired EEG band power (1–40 Hz) signals with a canonical correlation greater than the threshold, 0.99 (= param.ct in the function, 'rtcca'). Therefore, regularized Canonical Correlation Analysis with temporal embedding (tCCA) found fifteen regressors (shown in Additional file 1) from EEG band power (1–40 Hz) signals to reconstruct the corresponding fNIRS HbO signal from the LS channels using the GLM method in HOMER3 while regressing out the SS HbO signal representing systemic artefacts. The flowchart of the processing pipeline is shown in Fig. 3.

### Statistical analysis of the hemodynamic (HbO) response and the EEG microstates duration

The hemodynamic (HbO) response (10-s) during the FLS complex task and the error epoch was subjected to *t* test for each fNIRS channel to detect significant (*p* < 0.05) differences between experts and novices (i.e., skill level) after controlling for the false discovery rate (FDR). The Matlab function ‘hmrG_t_HRF_contrast2’ and ‘fdr_bh’ for *t* test and FDR are presented in the Additional file. Then, the visualization of the hemodynamic (HbO) response was performed using the AtlasViewer [1]. The Duration temporal property of the back fitted microstates, i.e., the proportion of the total time spent in each of the six microstates during the FLS complex task and the error epoch, was subjected to a two-way analysis of variance (ANOVA) with factors, skill level (expert, novice) and microstate types, after testing for normality with Shapiro Wilks Test. The significance level was set at α = 0.05.

## Results

**Fig. 4:**
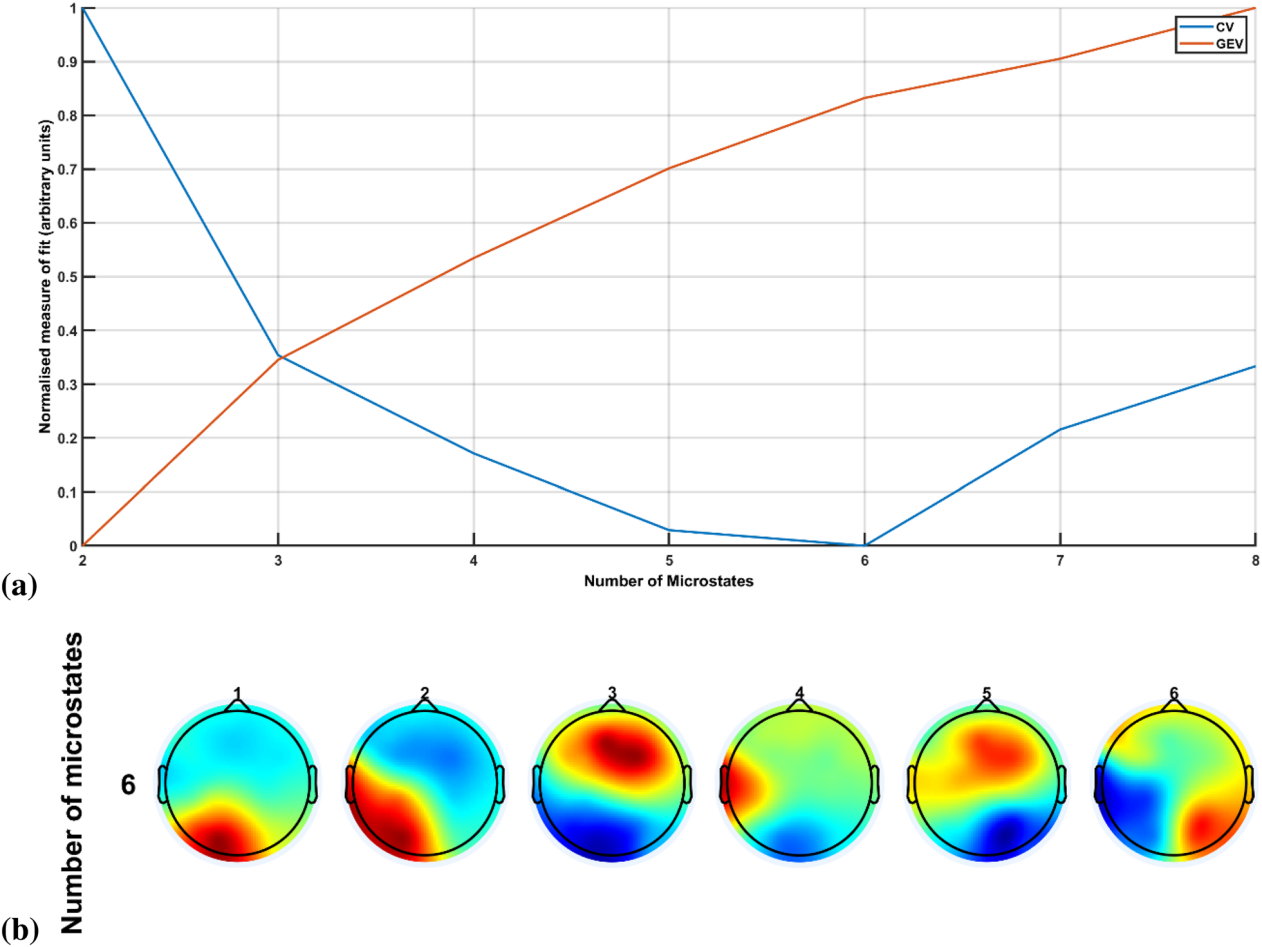
**a** Measures of fit plotted for the different microstate segmentations based on the global explained variance (GEV) and the cross-validation criterion (CV). **b** Selected six microstate prototypes based on the GEV and the CV criterion that are sorted in decreasing GEV

**Fig. 5:**
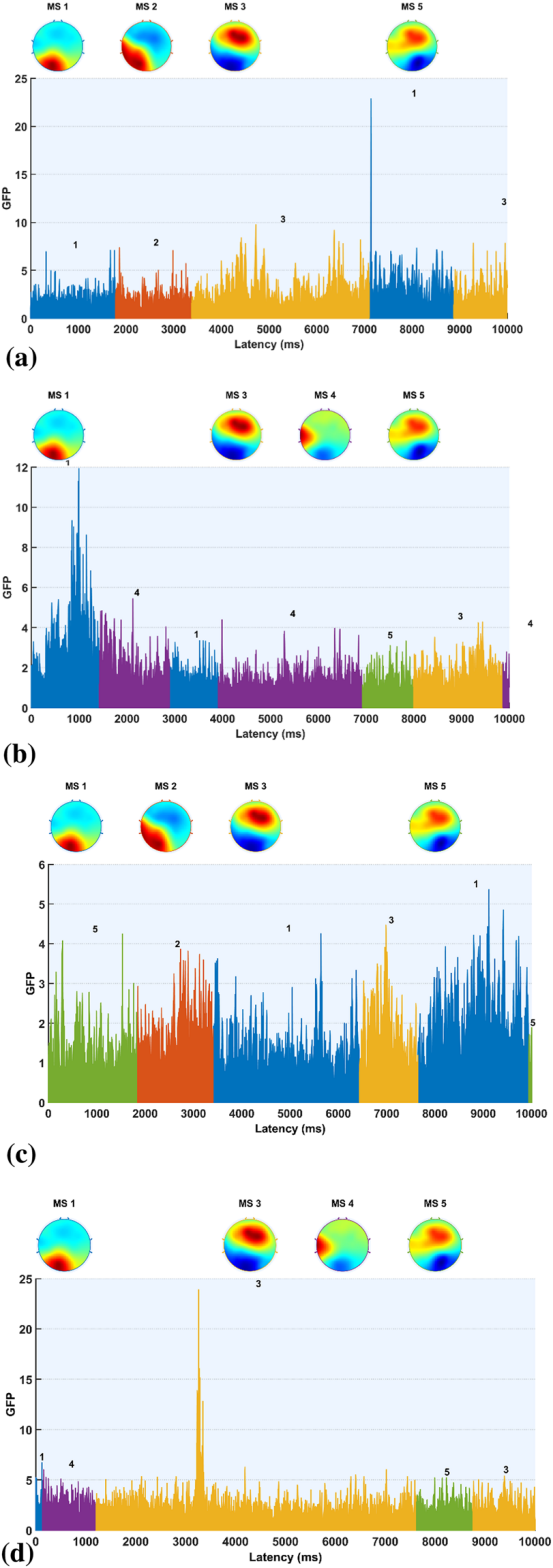

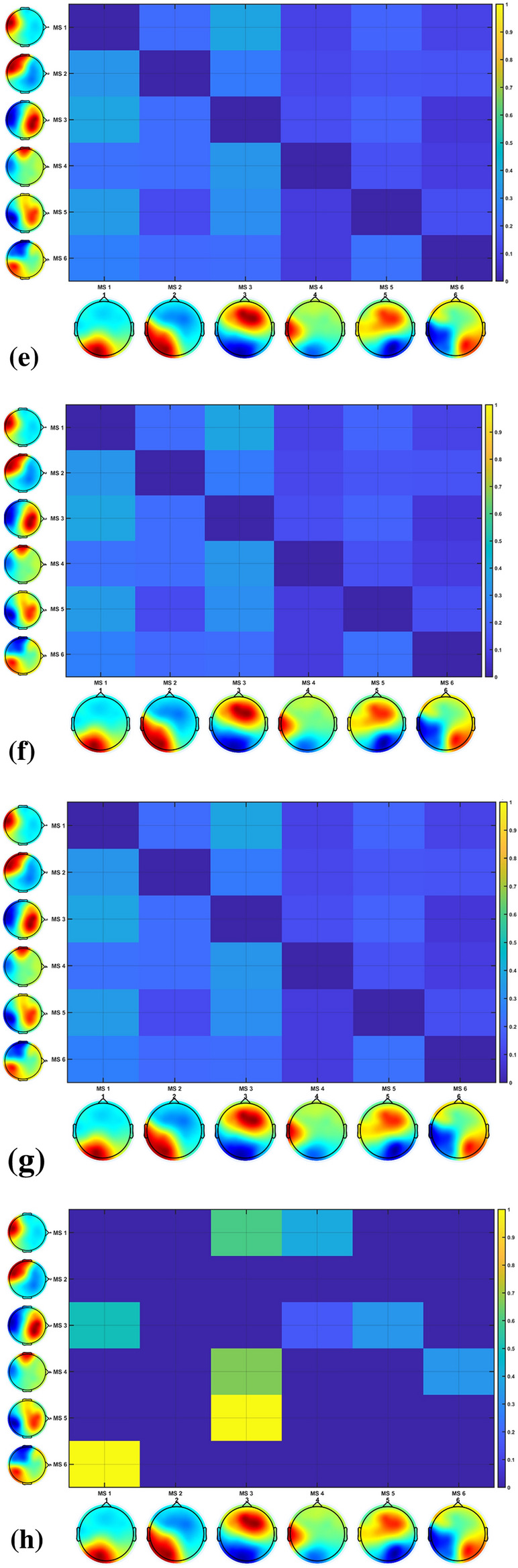
Illustrative figure of the GFP of active microstates dynamics, (**a**) during 0 to 10,000 ms at the start of the FLS complex task of the EEG of a novice, (**b**) during 0 to 10,000 ms at the start of the FLS complex task of the EEG of an expert, (**c**) during 10,000 ms of the error epoch of the EEG of a novice, (**d**) during 10,000 ms of the error epoch of the EEG of an expert. The statistics on the transition probabilities between microstate (MS) classes at the group level, (**e**) during the 10 s at the start of the FLS complex task in novices, (**f**) during the 10 s at the start of the FLS complex task in experts, (**g**) during the 10 s in the error epoch in novices, (**h**) during the 10 s in the error epoch in experts. In the transition probability matrix, the rows denote the ‘from’ microstate and the columns denote the ‘to’ microstate

We selected six EEG microstate prototypes based on the GEV and the CV criterion, as shown in Fig. 4a. The CV criterion, pointing to the best clustering solution at its smallest value, reached the minimum value for six microstates that explained 77.14% of the global variance. The six microstates in the decreasing order of their GEV, 18.96%, 15.71%, 14.64%, 9.84%, 9.77%, and 8.22%, are shown in Fig. 4b. The topographically similar microstates from Brechet and colleagues [9] are shown in the Additional file 1: Fig. S3. The six microstate prototypes were back fitted to the EEG for the 10-s duration at the start of the FLS complex task and during the error epoch, as shown in Fig. 5, for an expert and a novice.

The backfitting of the microstate prototypes (shown in Fig. 5b) to all the data points during 10 s at the start of the FLS complex task explained 64.29% GEV in novices and 73.64% GEV in the experts, while backfitting of the microstate prototypes to all the data points during 10 s in the error epoch explained 58.98% GEV in novices and 65.96% GEV in the experts. Figure 5a, b shows the GFP of the active states from 0 to 10000 ms at the start of the FLS complex task for a novice and expert, respectively, while Fig. 5c, d shows the GFP of the active states from 0 to 10000 ms during the error epoch of a novice and an expert, respectively. The first microstate at the start of the FLS complex task for a novice and expert is microstate 1. Then, microstate 2 was only present in the novice, while microstate 4 was only present in the expert during the initial 10 s of the FLS complex task. Then, the first 10 s of error processing-related brain states were captured in the expert (Fig. 5d) by microstate 1, followed by microstate 4, microstate 3, and microstate 5. At the same time, the novice (Fig. 5c) activated microstate 5 followed by microstate 2, microstate 1, and microstate 3 during the 10 s of error processing.

The statistical properties of the 6 microstates at the group level during the 10 s at the start of the FLS complex task for experts were, average GEV: 37.49%, 3.73%, 14.12%, 2.28%, 3.28%, 0.53%; average GFP: 2.73, 2.33, 2.18, 2.02, 1.99, 2.09; average spatial Correlation: 0.66, 0.54, 0.57, 0.49, 0.52, 0.45; Occurrence: 0.07, 0.05, 0.09, 0.05, 0.07, 0.02; Duration: 4566.58, 1862.80, 3512.15, 2103.03, 1791.79, 1485.93; Coverage: 0.34, 0.09, 0.33, 0.09, 0.12, 0.03. Here, microstate 1 accounted for the highest GEV during the 10 s at the start of the FLS complex task for experts. Then, the statistical properties of the 6 microstates at the group level during the 10 s at the start of the FLS complex task for novices were, average GEV: 0.10%, 5.49%, 15.72%, 4.16%, 8.74%, 4.22%; average GFP: 1.92, 2.15, 2.37, 2.81, 2.48, 2.41; average spatial Correlation 0.60, 0.56, 0.59, 0.48, 0.54, 0.48; Occurrence: 0.11, 0.07, 0.10, 0.04, 0.07, 0.04; Duration: 2712.02, 1944.020, 2582.80, 1898.35, 2056.34, 2020.87; Coverage: 0.29, 0.14, 0.27, 0.08, 0.14, 0.08. Here, microstate 3 accounted for the highest GEV during the 10 s at the start of the FLS complex task for novices. Then, the statistics on the transition probabilities between microstate classes during the 10 s at the start of the FLS complex task at the group level are shown in Fig. 5e, f for the novices and experts, respectively. Here, transition probabilities from microstate 1 to microstate 3 and from microstate 3 to microstate 1 (0.38 and 0.39, respectively) were top two in novices, while the transition probabilities from microstate 2 to microstate 3 and from microstate 5 to microstate 3 (0.41 and 0.40, respectively) were top two in experts (actual values are provided in the Additional file 1).

The statistical properties of the 6 microstates at the group level during the 10 s of the error epoch for experts were, average GEV: 11.25%, 0%, 25.16%, 1.37%, 8.81%, 2.89%; average GFP: 2.20, NaN, 2.75, 1.99, 3.82. 3.62; average spatial Correlation: 0.64, NaN, 0.57, 0.47, 0.57, 0.47; Occurrence: 0.10, 0, 0.14, 0.06, 0.04, 0.02; Duration: 2916.80, 0, 3601.14, 1496, 1716, 2268; Coverage: 0.29, 0, 0.50, 0.09, 0.07, 0.05. Here, microstate 2 is not present, while microstates 1 and 3 have the top two high GEV, with microstate 3 higher GEV than microstate 1, during the 10 s of the error epoch for experts. Then, the statistical properties of the 6 microstates at the group level during the 10 s of the error epoch for novices were, average GEV: 14.84%, 4.22%, 12.73%, 2.11%, 5.21%, 4.10%; average GFP: 2.54, 2.22, 2.88, 2.54, 2.46, 2.79; average spatial Correlation: 0.57, 0.56, 0.57, 0.48, 0.54, 0.46; Occurrence: 0.11, 0.09, 0.10, 0.03, 0.07, 0.07; Duration: 3112.73, 1500.44, 2021.60, 2496, 1880, 1624; Coverage: 0.34, 0.14, 0.20, 0.08, 0.13, 0.11. Here, microstates 1 and 3 have the top two high GEV, with microstate 1 higher GEV than microstate 3, during the 10-s error epoch for novices. Then, the statistics on the transition probabilities between microstate classes during the 10 s of the error epoch are shown in Fig. 5g, h for the novices and experts, respectively. Here, transition probabilities from microstate 3 to microstate 1 and from microstate 4 to microstate 1 (0.44 and 0.67, respectively) were the top two in novices, while the transition probabilities from microstate 5 to microstate 3 and from microstate 6 to microstate 1 (0.99 and 0.99, respectively) were top two in experts (actual values are provided in the Additional file 1).

A two-way ANOVA revealed that the proportion of the total time spent in the microstates during the 10-s duration at the start of the FLS complex task was statistically significantly affected by the skill level (F(1,120) = 7.58,*p* = 0.0068) and the interaction between the skill level and the microstate (F(5,120) = 2.51, *p* = 0.338). In addition, two-way ANOVA revealed that the proportion of the total time spent in microstates during the 10-s error epoch was statistically significantly affected by the skill level (F(1,120) = 22.29, *p* < 0.001), microstate (F(1,120) = 18.33, *p* < 0.001), and the interaction between the skill level and the microstate (F(5,120) = 30.66, *p* = 0.338).

**Fig. 6:**
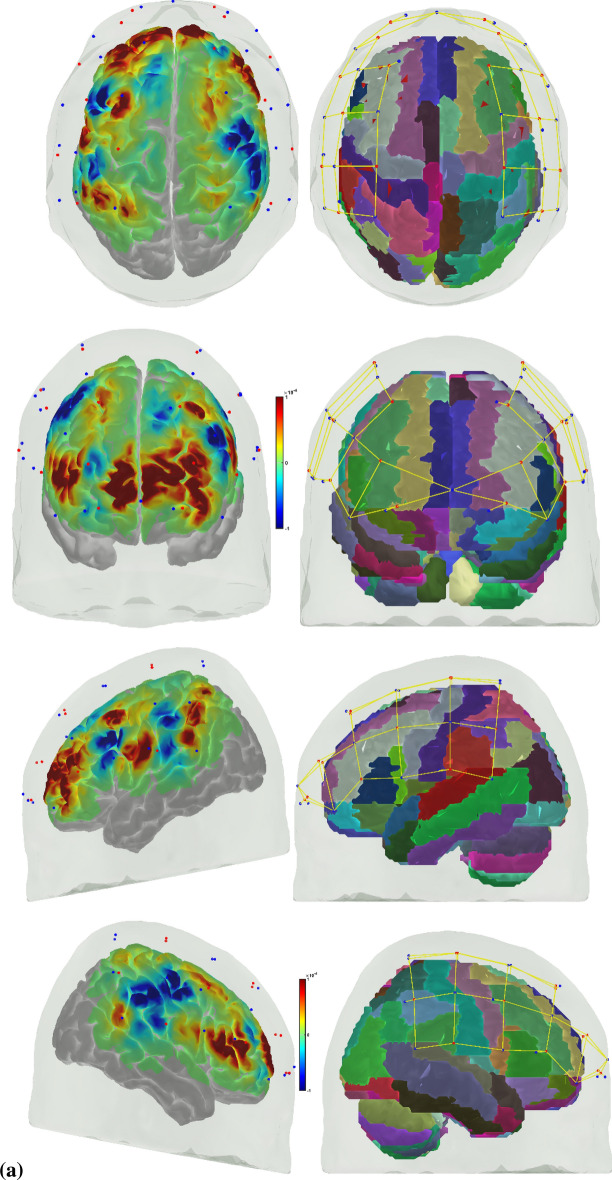

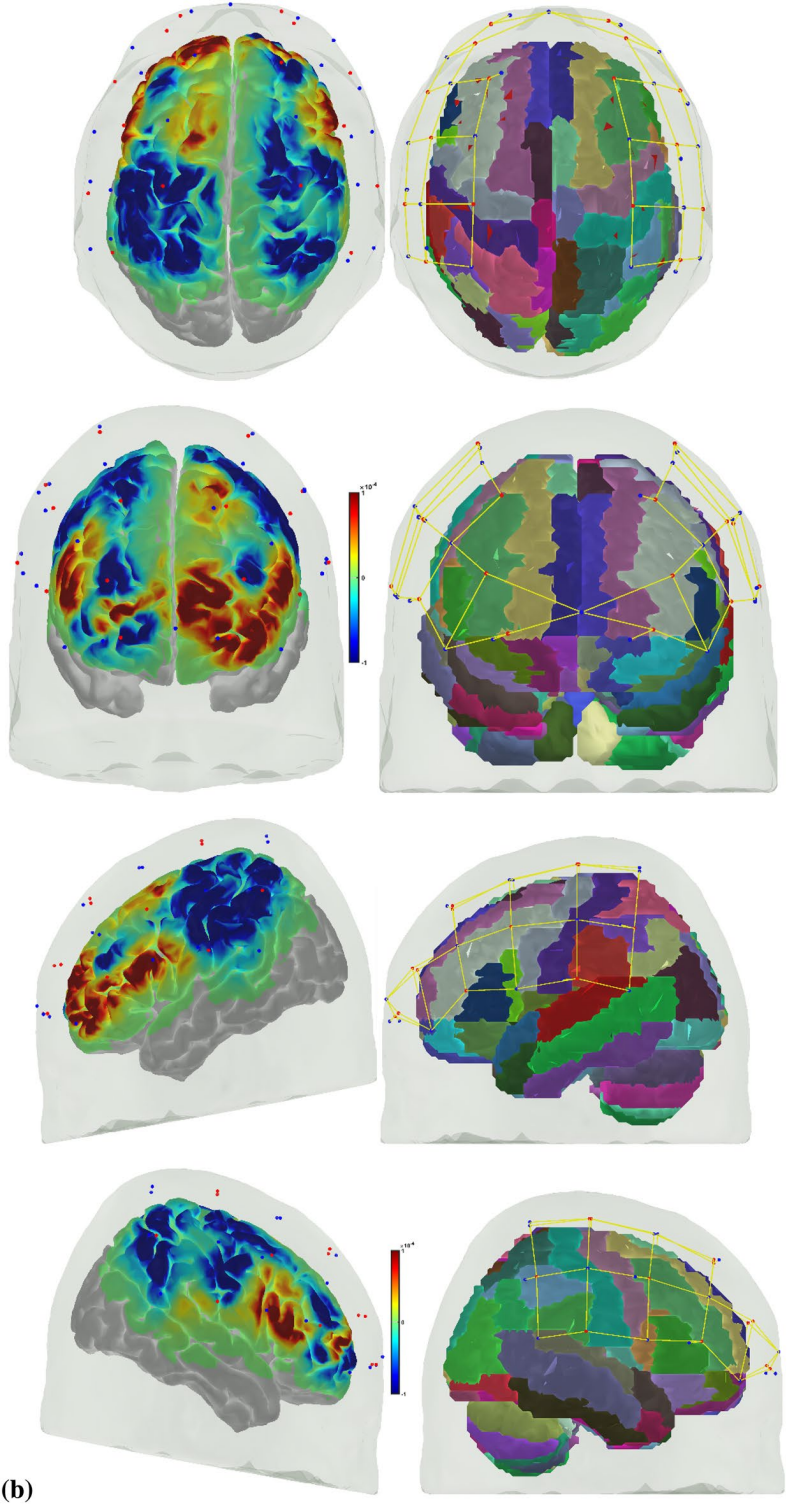

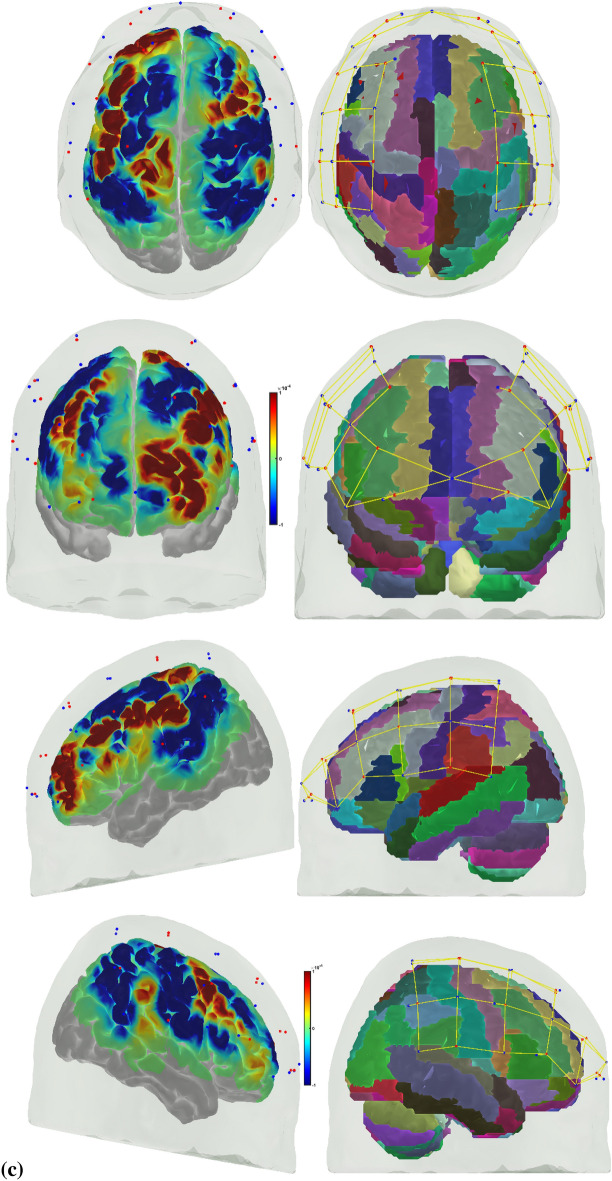

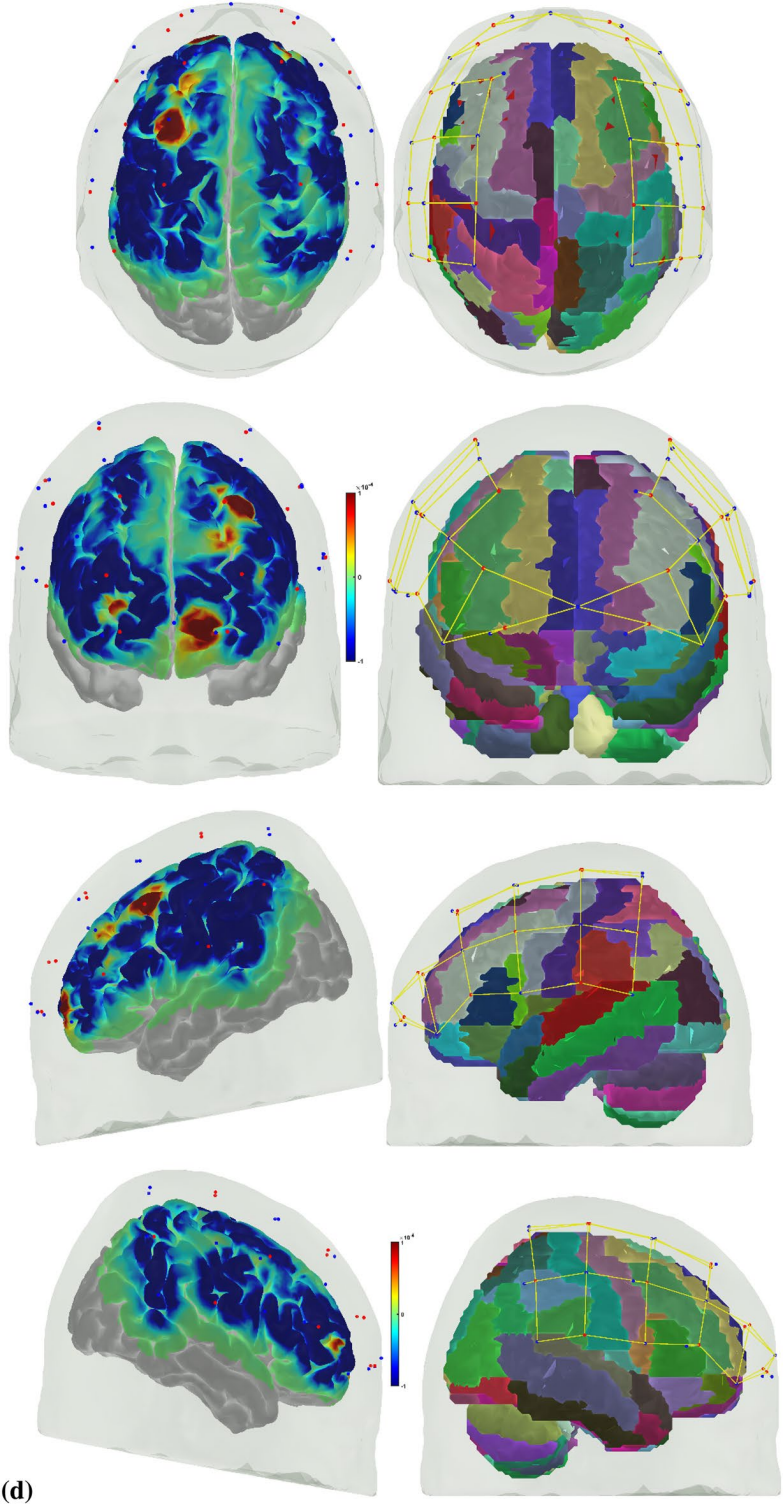
Image of the changes in HbO absorption coefficient in the cortex at the group level (left panel) along with the Automated Anatomical Labelling (AAL) of the brain regions in color (right panels). **A** During 0 to 10 s at the start of the FLS complex task of the novices, (**B**) during 0 to 10 s at the start of the FLS complex task of the experts, (**C**) during 0 to 10 s during the error epoch of the novices, (**D**) during 0 to 10 s during the error epoch of the experts

The image of the changes in the HbO absorption coefficient in the cortex was computed in the AtlasViewer [1] from the EEG band power (1–40 Hz)-related changes in the LS HbO signals using GLM with SS regression following regularized temporally embedded Canonical Correlation Analysis [96] in the HOMER3. The correlation coefficient of the GLM fit to the fNIRS data in HOMER3 (Huppert et al., 2009, 3) is shown in the Additional file 1: Tables S1 and S2 for novices and experts, respectively. Then, AtlasViewer [1] provided the image of the corresponding changes in the HbO absorption coefficient in the cortex during the 10-s duration at the start of the FLS complex task and during the error epoch that is shown in Fig. 6 for experts and novices at the group level.

While microstate prototypes were computed from EEG data with a high temporal resolution, the corresponding fNIRS (HbO) activity is a low-pass filtered version (under neurovascular coupling phenomenon) shown in Fig. 6 as changes in the HbO absorption coefficient in the cortex. A significant difference (*p* < 0.05) in the hemodynamic (HbO) response between the novices and the experts across fNIRS channels (listed in Table 1) during 0 to 10 s at the start of the FLS complex task and in the error epoch is shown in bold with ‘*’ in Table 2. During the FLS complex task, HbO signal from the fNIRS channels overlying left postcentral gyrus and right superior frontal gyrus—orbital part from AAL showed a significant (*p* < 0.05) difference, whereas HbO signal from the fNIRS channel overlying left inferior frontal gyrus—opercular part, left superior frontal gyrus—medial orbital, left postcentral gyrus, left superior temporal gyrus, right superior frontal gyrus—medial orbital part from AAL showed a significant (*p* < 0.05) difference in the error epoch.

**Table 2:** Difference (*p* value) in the hemodynamic (HbO) response across all fNIRS channels between the novices and the experts during 0 to 10 s at the start of the FLS complex task and in the error epoch. Automated anatomical labelling (AAL) of the cortical areas underlying fNIRS channels (source #–detector # pair) are also listed based on AtlasViewer's default head model [1]

Source #	Detector #	Regions	FLS complex task—*p* value	Error epoch—*p* value
1	1	Inferior frontal gyrus, orbital part left hemisphere	0.78	0.35
1	15	Superior frontal gyrus, orbital part left hemisphere	0.95	0.69
1	16	Superior frontal gyrus, orbital part left hemisphere	0.64	0.79
2	2	Superior frontal gyrus, dorsolateral left hemisphere	0.35	0.77
2	3	Middle frontal gyrus left hemisphere	0.59	0.78
2	17	Superior frontal gyrus, dorsolateral left hemisphere	0.45	0.17
3	1	Inferior frontal gyrus, orbital part left hemisphere	0.45	0.27
3	3	Middle frontal gyrus left hemisphere	0.17	0.67
3	5	Inferior frontal gyrus, opercular part left hemisphere	0.14	0.03*
4	1	Superior frontal gyrus, medial orbital left hemisphere	0.84	0.04*
4	3	Inferior frontal gyrus, triangular part left hemisphere	0.19	0.56
4	15	Superior frontal gyrus, orbital part left hemisphere	0.71	0.29
5	2	Middle frontal gyrus left hemisphere	0.59	0.54
5	3	Middle frontal gyrus left hemisphere	0.80	0.08
5	4	Postcentral gyrus left hemisphere	0.11	0.11
5	5	Precental gyrus left hemisphere	0.41	0.23
6	4	Postcentral gyrus left hemisphere	0.04^*^	0.04^*^
6	5	Superior temporal gyrus left hemisphere	0.37	0.01*
6	6	Rolandic operculum left hemisphere	0.77	0.44
6	18	Supramarginal gyrus left hemisphere	0.46	0.34
7	2	Superior frontal gyrus, dorsolateral left hemisphere	0.74	0.89
7	4	Postcentral gyrus left hemisphere	0.04^*^	0.48
7	7	Postcentral gyrus left hemisphere	0.18	0.42
8	4	Postcentral gyrus left hemisphere	0.16	0.23
8	6	Supramarginal gyrus left hemisphere	0.23	0.73
8	7	Inferior parietal, but supramarginal and angular gyri left hemisphere	0.14	0.44
8	19	Inferior parietal, but supramarginal and angular gyri left hemisphere	0.59	0.51
9	8	Middle frontal gyrus, orbital part right hemisphere	0.33	0.04^*^
9	15	Superior frontal gyrus, orbital part right hemisphere	0.04^*^	0.44
9	20	Superior frontal gyrus, dorsolateral right hemisphere	0.88	0.23
10	8	Inferior frontal gyrus, orbital part right hemisphere	0.34	0.11
10	9	Inferior frontal gyrus, triangular part right hemisphere	0.23	0.14
10	13	Inferior frontal gyrus, opercular part right hemisphere	0.80	0.09
10	21	Inferior frontal gyrus, triangular part right hemisphere	0.79	0.78
11	9	Superior frontal gyrus, dorsolateral right hemisphere	0.44	0.97
11	10	Superior frontal gyrus, dorsolateral right hemisphere	0.05	0.80
12	8	Middle frontal gyrus, orbital part right hemisphere	0.64	0.19
12	9	Middle frontal gyrus right hemisphere	0.32	0.15
12	15	Superior frontal gyrus, orbital part right hemisphere	0.47	0.32
13	9	Inferior frontal gyrus, triangular part right hemisphere	0.78	0.45
13	10	Precental gyrus right hemisphere	0.34	0.72
13	12	Precental gyrus right hemisphere	0.57	0.35
13	13	Precental gyrus right hemisphere	0.47	0.62
13	22	Precental gyrus right hemisphere	0.71	0.89
14	12	Supramarginal gyrus right hemisphere	0.34	0.59
14	13	Insula Right hemisphere	0.81	0.71
14	14	Superior temporal gyrus right hemisphere	0.40	0.77
15	10	Superior frontal gyrus, dorsolateral right hemisphere	0.17	0.45
15	11	Postcentral gyrus right hemisphere	0.05	0.54
15	12	Precental gyrus right hemisphere	0.85	0.14
16	11	Superior parietal gyrus right hemisphere	0.18	0.69
16	12	Supramarginal gyrus right hemisphere	0.83	0.86
16	14	Angular gyrus right hemisphere	0.59	0.82
16	23	Angular gyrus right hemisphere	0.57	0.23

## Discussion

In this study, EEG-based microstate analysis provided insights based on the changes in the scalp topography, as shown by an illustrative example of the GFP of the active microstate dynamics in Fig. 5. We analyzed the EEG microstates at the group level, since single-subject EEG microstate characteristics can be reliably unique while possessing abundant inter-individual variability [61]. Therefore, EEG-based microstate analysis needs to account for this inter-individual variability when discriminating experts from novices. At the group level, we found that microstates 1 and 3 were the most dominant (high GEV) across all conditions, and microstate 2 was found missing in the experts during 10 s at the error epoch. Microstate 2 is most topographically similar to Brechet and colleagues [9] microstate A (see Additional file 1: Fig. S3) that showed left-lateralized activity in the superior temporal gyrus (STG), the medial prefrontal cortex (MPFC), and the occipital gyri (OCG). In addition, microstate 2 is most topographically similar to Custo et al.’s [16] microstate A, which represents the left middle and superior temporal lobe activity, which is postulated to be associated with the exploration of both object-related and space-related information [47]. Here, the missing microstate 2 in experts indicated a lack of exploratory motor behavior in the error epoch. Then, during 10 s at the start of the FLS complex task, microstate 1 accounted for the highest GEV for experts, while microstate 3 accounted for the highest GEV for novices. In addition, during 10 s at the error epoch, microstate 3 accounted for the highest GEV for experts, while microstate 1 accounted for the highest GEV for novices. Microstate 3 is topographically similar to Brechet and colleagues [9] microstate D (see Additional file 1: Fig. S3), where the sources showed main activity bilaterally in the inferior frontal gyrus (IFG), dorsal anterior cingulate cortex (dACC), and superior parietal lobule (SPL)/intraparietal sulcus (IPS). Then, microstate 1 is most topographically similar to Brechet and colleagues [9] microstate C (see Additional file 1: Fig. S3), where the sources showed sources located bilaterally in the lateral part of the parietal cortex, including both the supramarginal gyrus (SMG) and angular gyrus (AG). Here, we postulate that the activation of SMG and AG during the FLS complex task indicated ventral attention, while the activation of SPL/IPS indicated the dorsal attention systems relevant to reorienting of visuospatial attention [98] from perception to action. ANOVA revealed a significant effect of skill level (expert, novice) on the proportion of the total time spent in the microstates during the 10-s duration at the start of the FLS complex task and error epoch. Microstate 3 can also be topographically related to the canonical microstate D from the combined EEG–fMRI recording of the resting state published by Britz et al. [10]. Microstate D, published by Britz et al. [10], was shown by a behavioral manipulation study by Milz et al. [66] that reflected reflexive aspects of attention, focus switching, and reorientation [64], which is necessary for error-related switching of the mental state.

The HbO response and the image of the average changes in HbO absorption coefficient in the cortex during the 10-s epoch are shown in Fig. 6. Here, HbO response captured the slower hemodynamic activity of the brain due to neurovascular coupling during a longer 10-s epoch. Statistical testing of the HbO hemodynamic response at the fNIRS channels identified underlying left postcentral gyrus and right superior frontal gyrus (SFG)—orbital part as significantly different between the experts and novices during 10 s at the start of the FLS complex task, while HbO hemodynamic response at the underlying left IFG—opercular part, left SFG—medial orbital, left postcentral gyrus, left STG, right SFG—medial orbital were significantly different between experts and novices during the error epoch. Here, the postcentral gyrus contains the primary somatosensory cortex, and the right SFG—orbital part contributes to the proactive control of the impulses [42] relevant in the performance of the FLS complex task that was different between experts and novices. In addition, the activation of the left IFG—opercular part, left SFG—medial orbital can be related to higher cognitive functions [21], while the activation of the right SFG—medial orbital can be related to the proactive control of the impulses [42] both relevant in error processing that was different between experts and novices. Moreover, while the left postcentral gyrus is related to the motor action in the right-handed subjects, the STG can be related to the exploration [47] during error processing that was different between experts and novices, viz., we found microstate 2 that is related to the left middle and superior temporal lobe [16] missing in the experts. We showed the fusion of information from simultaneously acquired EEG and fNIRS signals to provide mechanistic insight into the changes in the brain state during FLS complex task and error perception/correction during FLS skill training. Notably, Fig. 6d shows the hemodynamic response during 0 to 10 s during the error epoch of the experts demonstrated a global suppressive effect [29] that excluded left-hemispheric frontopolar and dorsolateral prefrontal/frontal eye field brain regions. Such global suppressive effects can be associated with arousal-related cortical activity [6] that needs future investigation, e.g., using pupillometry [63]. Specifically, the locus coeruleus norepinephrine (LC-NE) arousal system has strong projections to cortex for the modulation of the visual attention [86], where a ‘focusing’ LC-NE effect on the hemodynamics for the fronto-parietal networks in skilled experts can be postulated [36]. The video data from our study showed that the experts primarily had "incorrect needle insertion" error events that were skillfully stopped as soon as the needle emerged incorrectly from the Penrose drain, i.e., fast sensorimotor inhibition on error visibility.

Statistics on the transition probabilities between microstate classes at the group level showed that the novices mostly transitioned between microstate 3 to microstate 1 during the 10 s at the start of the FLS complex task and from microstates 3,4 to microstate 1 in 10 s of the error epoch, where microstate 1 can be associated with posterior isoelectric point in the topographical map of the salience network [83] that is involved in attending to and responding to error (unexpected) stimuli. Here, microstate 4 is most topographically similar to Brechet and colleagues [9] microstate F (see Additional file 1: Fig. S3) showed the strongest activity in the right MPFC. In contrast, the experts mainly transitioned from microstates 2,5 to microstate 3 during the 10 s at the start of the FLS complex task and from microstate 5 to microstate 3, from microstate 6 to microstate 1 in the 10 s of the error epoch. Here, microstate 5 is topographically similar to Brechet and colleagues [9] microstate F (see Additional file 1: Fig. S3) shows bilateral activity in the MPFC. Then, microstate 6 is most topographically similar to Brechet and colleagues [9] microstate B (see Additional file 1: Fig. S3) showed main activity in OCG and in the medial part of the parietal cortex. Microstate 6 can be associated with spatial attention [10], so the transition to microstate 1 of the salience network [83] highlights visual error awareness and salience processing of error (unexpected) stimuli in the experts. Moreover, the transition of microstates 1,4,5 to microstate 3 with high (> 0.6, see Additional file 1: Fig. S2) transition probabilities illustrate the learned reflexive aspects of attention, focus switching, and reorientation [64] in the experts during error-related adjustments. Here, the microstate correlates of exploration–exploitation tradeoff, e.g., microstate 2 for motor exploration [47] and microstate 3 with SPL activity for planning and guiding movement relevant to motor exploitation, are postulated to differentiate experts and novices [72] during error-related adjustments that need further investigation in the future studies. Moreover, the microstate transitions can be related to a cortical traveling wave [67] subserving the hierarchical sequencing of local brain regions in the perception action system (Fig. 1a) that needs further investigation in the future.

Our brain activation results aligned with numerous functional magnetic resonance imaging (fMRI) and fNIRS studies that have been published on skill learning [30, 31, 50, 56–58, 68, 80, 101]. Published fMRI studies have shown that a large-scale brain network encodes motor learning and transfer of learning from related past experiences [33, 40]. The prefrontal cortex (PFC) has been found to integrate the information necessary for action generation and perception (Raos and Savaki, 2017) relevant to error processing during FLS task performance. Specifically, FLS task performance is graded based on the speed and accuracy of the psychomotor skills [79], where speed–accuracy tradeoff during skill training can lead to automaticity when there is a greater focus on speed despite the residual error, i.e., an increased speed of action selection at the cost of cognitive flexibility [74, 94] affecting error processing. Indeed, not everyone can achieve proficiency [35], and we postulate that successful skill acquisition needs cognitive flexibility [74, 94] for error-based motor learning despite a post-error slowing of action selection [85]. Here, successful skill acquisition leads to an internal forward model [104] that can simulate the perceptual consequences of the planned and executed motor commands. An intact action–perception coupling has been shown to depend on the integrity of the cerebellum [12] that underpins the internal model [22] and error-based learning [75]. Error-based sensorimotor learning also involves other brain areas, including the parietal cortex, striatum, and anterior cingulate cortex [85]. Then, the hierarchy of the cognitive control during skill learning shows a rostrocaudal axis in the frontal lobe [5]), where a shift from posterior to anterior is postulated to mediate progressively abstract, higher order control expected with skill learning. In this study, the dorsolateral and ventrolateral PFC showed activation in Fig. 6a, b during the FLS complex task that can be related to attention control, cognitive control, feature extraction, and formation of first-order relationships [4, 5, 13, 51]. Specifically, the dorsolateral PFC of the dorsal stream is more involved in the visual guidance of action in novices (see Fig. 6a) relevant in motor exploration [87]. In contrast, the ventrolateral PFC of the ventral stream is more involved in the recognition and conscious perception [65] in experts (see Fig. 6b) relevant to motor exploitation [87]. Then, the supplementary motor area (SMA) and the premotor cortex are crucial for the coordination of bimanual movement [93], where SMA is crucial for complex spatiotemporal sequencing of movements [18, 92] necessary in bimanual FLS complex task. In addition, the cingulate and pre-supplementary motor areas are the generator sites of error-related negativity that is time-locked to an erroneous response [85]. Here, the medial frontal cortex is known to serve a central role in performance monitoring [26] that is crucial for cognitive flexibility. In this study, the dACC activity was captured by microstate 3, one of the most dominant (high GEV) microstates across all conditions.

Future study needs to combine pupillometry with EEG microstate analysis which may elucidate the relationship of microstate 3 with the error-related pupil dilation or constriction during skill training vis-à-vis the significance of errors for adaptive behavioral adjustments [62]. Then, SMA is involved in planning complex motor sequence of finger tasks [69] that are critical in error correction [85]. Here, EEG microstates, e.g., related to the canonical subjective interoceptive–autonomic processing [10], may be a marker of error-specific autonomic arousal mechanisms that promote post-error adjustments [62] differentially in fast versus slow learners. Then, brain–behavior monitoring of the error-related cortical activation and corrective action can allow appropriate error feedback for operant conditioning in future work that has been shown feasible in our prior application for stroke rehabilitation [52]. For example, novices may lack error perception (e.g., lack of medial frontal cortex activation on errors [32]) that can disrupt their skill learning, which can be improved with non-invasive brain stimulation of the medial frontal cortex in conjunction with explicit error feedback in the medical simulator. Then, EEG topographies provide subject-specific correlates of motor control [73], where portable neuroimaging guided non-invasive brain stimulation may be feasible [99] to enforce beneficial scalp topographies to facilitate perception and action that together form a functional system. The two crucial attributes of the perception–action cycle are perceptual, and executive memory [28], and error sensitivity is postulated to depend on the memory of errors, i.e., the history of past consistent perceptual errors, e.g., error in depth prediction from a 2D view [75] or executive errors, e.g., “incorrect needle insertion” [2]. Then, early efferent error prediction can lead to preemptive adjustments in experts who know the action semantics, e.g., skilled typists execute errors with lighter keystrokes than novices. Published studies have shown that the pre-supplementary motor area (pre-SMA) and the inferior frontal gyrus are involved in stop-signal task performance [85] that is necessary for immediate error-related adjustments. In addition, published fNIRS studies showed the involvement of the inferior parietal cortex, PFC, occipital cortex, and the sensorimotor areas, including the premotor and primary motor cortex, during skill training. In contrast, the fMRI studies showed additional activation of deeper brain structures, including the basal ganglia and cerebellum [80]. Future studies need to apply the perception–action system based on brain–behavior analysis during a learning curve study [25], where the chain of mental processes can depend on the task complexity [100], 707).

It is known from skill training studies that the hierarchy of cognitive control shows a rostrocaudal axis in the frontal lobe, where a shift from posterior to anterior is postulated to mediate progressively abstract, higher order control. The current study used portable fNIRS with limited spatial and depth sensitivity (Strangman et al., 2013), so it could provide a partial view of the brain network. Therefore, the main limitation of our study includes a low-density fNIRS and EEG sensor montage that limited the spatial resolution to capture the complete hierarchy, as shown in Fig. 1a. Our multimodal imaging approach also limited the head cap space for each of the modalities due to separate optodes and electrodes in the sensor montage, where an integrated "co-located" optode + electrode (optrode) can be helpful [49] for high-density brain imaging in the future.

## Conclusion

We conclude that the error-related chain of mental processes differs between experts and novices during the FLS intracorporeal suturing and knot tying task that can be associated with the contextual switching of the brain states on error commission. Specifically, novices did not demonstrate any prominent microstate transition probabilities (top two 0.44 and 0.67 only) in the 10-s error epoch, whereas experts showed dominant microstate transition probabilities (top two 0.99 and 0.99) that can be associated with visual error awareness and salience processing of error (unexpected) stimuli in the 10-s error epoch. Furthermore, experts demonstrated HbO activation of controlled voluntary attention-related brain areas, including the left dorsolateral prefrontal/frontal eye field and left frontopolar brain regions, along with global suppressive effects of the sensorimotor areas. In contrast, the novices showed widespread error-driven activation of the frontoparietal and the sensorimotor areas that are postulated to be involuntary.

## Supplementary Information

**Additional file 1: Figure S1.** Illustrative plot of the 15 components or sources (greater than the correlation threshold, 0.99) in the tCCA latent space, where red are the EEG bandpower (1–40Hz) sources and the black lines are the corresponding HbO sources. The 15 EEG sources were used as the regressors along with short-separation nuisance regressors in the GLM to reconstruct the HbO signal. **Figure S2.** Statistics on the transition probabilities between microstate (MS) classes at the group level, (A) during the 10 s at the start of the FLS complex task in novices, (B) during the 10 s at the start of the FLS complex task in experts, (C) during the 10 s in the error epoch in novices, (D) during the 10 s in the error epoch in experts. The rows denote the ‘from’ microstate and the columns denote the ‘to’ microstate. **Figure S3.** Six microstate prototypes shown in the top row and the topographically similar microstates from Brechet and colleagues (Bréchet et al., 2019) shown in the bottom row. In Brechet and colleagues (Bréchet et al., 2019), microstate A showed left-lateralized activity in the superior temporal gyrus (STG), the medial prefrontal cortex (MPFC) and the occipital gyri (OCG). Microstate B showed main activity in OCG and in the medial part of the parietal cortex. The sources of microstate C were located bilaterally in the lateral part of the parietal cortex including both the supramarginal gyrus (SMG) and angular gyrus (AG). The sources of microstate D showed main activity bilaterally in the inferior frontal gyrus (IFG), dorsal anterior cingulate cortex (dACC), and superior parietal lobule (SPL)/intraparietal sulcus (IPS). Strongest activity for microstate E was found in the right MPFC. Finally, microstate F showed bilateral activity in the MPFC. **Table S1.** R—the correlation coefficient of the GLM fit to the data (#Channels x HbO) in HOMER3 for the novices, N01–N13. **Table S2.** R—the correlation coefficient of the GLM fit to the data (#Channels x HbO) in HOMER3 for the experts, E01–E09.

## Data Availability

Data available on request due to privacy/ethical restrictions.
