# Supplementary material for: Error-related brain state analysis using electroencephalography in conjunction with functional near-infrared spectroscopy during a complex surgical motor task
Source: Brain Inform. 2022 Dec 9;9(1):29. doi: 10.1186/s40708-022-00179-z (PMC9733771; doi:10.1186/s40708-022-00179-z)
Supplement: Supplementary file 1 — Additional file 1: Figure S1. Illustrative plot of the 15 components or sources (greater than the correlation threshold, 0.99) in the tCCA latent space, where red are the EEG bandpower (1–40Hz) sources and the black lines are the corresponding HbO sources. The 15 EEG sources were used as the regressors along with short-separation nuisance regressors in the GLM to reconstruct the HbO signal. Figure S2. Statistics on the transition probabilities between microstate (MS) classes at the group level, (A) during the 10 s at the start of the FLS complex task in novices, (B) during the 10 s at the start of the FLS complex task in experts, (C) during the 10 s in the error epoch in novices, (D) during the 10 s in the error epoch in experts. The rows denote the ‘from’ microstate and the columns denote the ‘to’ microstate. Figure S3. Six microstate prototypes shown in the top row and the topographically similar microstates from Brechet and colleagues (Bréchet et al., 2019) shown in the bottom row. In Brechet and colleagues (Bréchet et al., 2019), microstate A showed left-lateralized activity in the superior temporal gyrus (STG), the medial prefrontal cortex (MPFC) and the occipital gyri (OCG). Microstate B showed main activity in OCG and in the medial part of the parietal cortex. The sources of microstate C were located bilaterally in the lateral part of the parietal cortex including both the supramarginal gyrus (SMG) and angular gyrus (AG). The sources of microstate D showed main activity bilaterally in the inferior frontal gyrus (IFG), dorsal anterior cingulate cortex (dACC), and superior parietal lobule (SPL)/intraparietal sulcus (IPS). Strongest activity for microstate E was found in the right MPFC. Finally, microstate F showed bilateral activity in the MPFC. Table S1. R—the correlation coefficient of the GLM fit to the data (#Channels x HbO) in HOMER3 for the novices, N01–N13. Table S2. R—the correlation coefficient of the GLM fit to the data (#Channels x HbO) in HOMER3 for [file 40708_2022_179_MOESM1_ESM.docx]

Supplementary Materials – code for statistical tests in Matlab

% SYNTAX:

% [hmrstatsG_cond] = **hmrG_t_HRF_contrast2**(yAvgSubjs1, yAvgSubjs2, tHRFrange)

%

% UI NAME:

% t-test

%

% DESCRIPTION:

% Performs a t-test between two mean HRF for a single condition across all subjects

%

% INPUTS:

% yAvgSubjs: yAvgSubjs1 for group 1 and yAvgSubjs2 for group 2

% tHRFrange: tHRF range for HRF averaging

%

% OUTPUTS:

% hmrstatsG_cond: Statistical results from the MATLAB ttest (h,p,c,stats) and measurement list (ml)

%

% USAGE OPTIONS:

% Stats_on_Concentration_Data: [hmrstatsG_base_cond] = hmrG_t_HRF_contrast2(dcAvgSubjs1, dcAvgSubjs2, tHRFrange)

%

% PARAMETERS:

% tHRFrange: [0, 0]

%

function [hmrstatsG_cond] = hmrG_t_HRF_contrast2(yAvgSubjs1, yAvgSubjs2, tHRFrange)

hmrstatsG_base_cond = [];

iBlk=1;

nSubj = length(yAvgSubjs1);

for iSubj = 1:nSubj

yAvg = yAvgSubjs1{iSubj}(iBlk).GetDataTimeSeries('reshape');

ncond = size(yAvg,4);

if iSubj == 1

tHRF = yAvgSubjs1{iSubj}(iBlk).GetTime();

fq = abs(1/(tHRF(1)-tHRF(2)));

ml = yAvgSubjs1{iSubj}(iBlk).GetMeasListSrcDetPairs();

% error check

if tHRFrange(1)>max(tHRF) || tHRFrange(2)>max(tHRF) || tHRFrange(1)>=tHRFrange(2)

warning('tHRF range should be between 0 and tHRF max');

return

end

end

baseline_yAvg1(iSubj,:,:,:) = squeeze(mean(yAvg(1:round(fq*abs(min(tHRF))),:,:,:),1));

mean_yAvg1(iSubj,:,:,:) = squeeze(mean(yAvg(round(fq*(tHRFrange(1) + abs(min(tHRF)))):round(fq*(tHRFrange(2) + abs(min(tHRF)))),:,:,:),1));

mean_yAvg1(iSubj,:,:,:) = mean_yAvg1(iSubj,:,:,:) - baseline_yAvg1(iSubj,:,:,:);

end

nSubj = length(yAvgSubjs2);

for iSubj = 1:nSubj

yAvg = yAvgSubjs2{iSubj}(iBlk).GetDataTimeSeries('reshape');

ncond = size(yAvg,4);

if iSubj == 1

tHRF = yAvgSubjs2{iSubj}(iBlk).GetTime();

fq = abs(1/(tHRF(1)-tHRF(2)));

ml = yAvgSubjs2{iSubj}(iBlk).GetMeasListSrcDetPairs();

% error check

if tHRFrange(1)>max(tHRF) || tHRFrange(2)>max(tHRF) || tHRFrange(1)>=tHRFrange(2)

warning('tHRF range should be between 0 and tHRF max');

return

end

end

baseline_yAvg2(iSubj,:,:,:) = squeeze(mean(yAvg(1:round(fq*abs(min(tHRF))),:,:,:),1));

mean_yAvg2(iSubj,:,:,:) = squeeze(mean(yAvg(round(fq*(tHRFrange(1) + abs(min(tHRF)))):round(fq*(tHRFrange(2) + abs(min(tHRF)))),:,:,:),1));

mean_yAvg2(iSubj,:,:,:) = mean_yAvg2(iSubj,:,:,:) - baseline_yAvg2(iSubj,:,:,:);

end

% get t-stats

for iCond = 1:ncond

for i = 1:size(yAvg, 2) % HbO/R/T

for j = 1:size(yAvg,3) % Channels

[h,p,c,stats] = ttest2(mean_yAvg1(:,i,j,iCond),mean_yAvg2(:,i,j,iCond));

pval(i,j,iCond) = p;

hval(i,j,iCond) = h;

cval(i,j,iCond,:) = c;

tstats{i,j,iCond} = stats;

end

end

end

% output

hmrstatsG_cond.pval = pval;

hmrstatsG_cond.hval = hval;

hmrstatsG_cond.cval = cval;

hmrstatsG_cond.tstats = tstats;

hmrstatsG_cond.ml = ml;

hmrstatsG_cond.mean_yAvg1 = mean_yAvg1;

hmrstatsG_cond.mean_yAvg2 = mean_yAvg2;

% fdr_bh() - Executes the Benjamini & Hochberg (1995) and the Benjamini &

% Yekutieli (2001) procedure for controlling the false discovery

% rate (FDR) of a family of hypothesis tests. FDR is the expected

% proportion of rejected hypotheses that are mistakenly rejected

% (i.e., the null hypothesis is actually true for those tests).

% FDR is a somewhat less conservative/more powerful method for

% correcting for multiple comparisons than procedures like Bonferroni

% correction that provide strong control of the family-wise

% error rate (i.e., the probability that one or more null

% hypotheses are mistakenly rejected).

%

% This function also returns the false coverage-statement rate

% (FCR)-adjusted selected confidence interval coverage (i.e.,

% the Coverage needed to construct multiple comparison corrected

% confidence intervals that correspond to the FDR-adjusted p-values).

%

%

% Usage:

% >> [h, crit_p, adj_ci_cvrg, adj_p]=fdr_bh(pvals,q,method,report);

%

% Required Input:

% pvals - A vector or matrix (two dimensions or more) containing the

% p-value of each individual test in a family of tests.

%

% Optional Inputs:

% q - The desired false discovery rate. {default: 0.05}

% method - ['pdep' or 'dep'] If 'pdep,' the original Bejnamini & Hochberg

% FDR procedure is used, which is guaranteed to be accurate if

% the individual tests are independent or positively dependent

% (e.g., Gaussian variables that are positively correlated or

% independent). If 'dep,' the FDR procedure

% described in Benjamini & Yekutieli (2001) that is guaranteed

% to be accurate for any test dependency structure (e.g.,

% Gaussian variables with any covariance matrix) is used. 'dep'

% is always appropriate to use but is less powerful than 'pdep.'

% {default: 'pdep'}

% report - ['yes' or 'no'] If 'yes', a brief summary of FDR results are

% output to the MATLAB command line {default: 'no'}

%

%

% Outputs:

% h - A binary vector or matrix of the same size as the input "pvals."

% If the ith element of h is 1, then the test that produced the

% ith p-value in pvals is significant (i.e., the null hypothesis

% of the test is rejected).

% crit_p - All uncorrected p-values less than or equal to crit_p are

% significant (i.e., their null hypotheses are rejected). If

% no p-values are significant, crit_p=0.

% adj_ci_cvrg - The FCR-adjusted BH- or BY-selected

% confidence interval coverage. For any p-values that

% are significant after FDR adjustment, this gives you the

% proportion of Coverage (e.g., 0.99) you should use when generating

% confidence intervals for those parameters. In other words,

% this allows you to correct your confidence intervals for

% multiple comparisons. You can NOT obtain confidence intervals

% for non-significant p-values. The adjusted confidence intervals

% guarantee that the expected FCR is less than or equal to q

% if using the appropriate FDR control algorithm for the

% dependency structure of your data (Benjamini & Yekutieli, 2005).

% FCR (i.e., false coverage-statement rate) is the proportion

% of confidence intervals you construct

% that miss the true value of the parameter. adj_ci=NaN if no

% p-values are significant after adjustment.

% adj_p - All adjusted p-values less than or equal to q are significant

% (i.e., their null hypotheses are rejected). Note, adjusted

% p-values can be greater than 1.

%

%

% References:

% Benjamini, Y. & Hochberg, Y. (1995) Controlling the false discovery

% rate: A practical and powerful approach to multiple testing. Journal

% of the Royal Statistical Society, Series B (Methodological). 57(1),

% 289-300.

%

% Benjamini, Y. & Yekutieli, D. (2001) The control of the false discovery

% rate in multiple testing under dependency. The Annals of Statistics.

% 29(4), 1165-1188.

%

% Benjamini, Y., & Yekutieli, D. (2005). False discovery rate?adjusted

% multiple confidence intervals for selected parameters. Journal of the

% American Statistical Association, 100(469), 71?81. doi:10.1198/016214504000001907

%

%

% Example:

% nullVars=randn(12,15);

% [~, p_null]=ttest(nullVars); %15 tests where the null hypothesis

% %is true

% effectVars=randn(12,5)+1;

% [~, p_effect]=ttest(effectVars); %5 tests where the null

% %hypothesis is false

% [h, crit_p, adj_ci_cvrg, adj_p]=fdr_bh([p_null p_effect],.05,'pdep','yes');

% data=[nullVars effectVars];

% fcr_adj_cis=NaN*zeros(2,20); %initialize confidence interval bounds to NaN

% if ~isnan(adj_ci_cvrg),

% sigIds=find(h);

% fcr_adj_cis(:,sigIds)=tCIs(data(:,sigIds),adj_ci_cvrg); % tCIs.m is available on the

% %Mathworks File Exchagne

% end

%

%

% For a review of false discovery rate control and other contemporary

% techniques for correcting for multiple comparisons see:

%

% Groppe, D.M., Urbach, T.P., & Kutas, M. (2011) Mass univariate analysis

% of event-related brain potentials/fields I: A critical tutorial review.

% Psychophysiology, 48(12) pp. 1711-1725, DOI: 10.1111/j.1469-8986.2011.01273.x

% http://www.cogsci.ucsd.edu/~dgroppe/PUBLICATIONS/mass_uni_preprint1.pdf

%

%

% For a review of FCR-adjusted confidence intervals (CIs) and other techniques

% for adjusting CIs for multiple comparisons see:

%

% Groppe, D.M. (in press) Combating the scientific decline effect with

% confidence (intervals). Psychophysiology.

% http://biorxiv.org/content/biorxiv/early/2015/12/10/034074.full.pdf

%

%

% Author:

% David M. Groppe

% Kutaslab

% Dept. of Cognitive Science

% University of California, San Diego

% March 24, 2010

%%%%%%%%%%%%%%%% REVISION LOG %%%%%%%%%%%%%%%%%

%

% 5/7/2010-Added FDR adjusted p-values

% 5/14/2013- D.H.J. Poot, Erasmus MC, improved run-time complexity

% 10/2015- Now returns FCR adjusted confidence intervals

function [h, crit_p, adj_ci_cvrg, adj_p]=fdr_bh(pvals,q,method,report)

if nargin<1,

error('You need to provide a vector or matrix of p-values.');

else

if ~isempty(find(pvals<0,1)),

error('Some p-values are less than 0.');

elseif ~isempty(find(pvals>1,1)),

error('Some p-values are greater than 1.');

end

end

if nargin<2,

q=.05;

end

if nargin<3,

method='pdep';

end

if nargin<4,

report='no';

end

s=size(pvals);

if (length(s)>2) || s(1)>1,

[p_sorted, sort_ids]=sort(reshape(pvals,1,prod(s)));

else

%p-values are already a row vector

[p_sorted, sort_ids]=sort(pvals);

end

[dummy, unsort_ids]=sort(sort_ids); %indexes to return p_sorted to pvals order

m=length(p_sorted); %number of tests

if strcmpi(method,'pdep'),

%BH procedure for independence or positive dependence

thresh=(1:m)*q/m;

wtd_p=m*p_sorted./(1:m);

elseif strcmpi(method,'dep')

%BH procedure for any dependency structure

denom=m*sum(1./(1:m));

thresh=(1:m)*q/denom;

wtd_p=denom*p_sorted./[1:m];

%Note, it can produce adjusted p-values greater than 1!

%compute adjusted p-values

else

error('Argument ''method'' needs to be ''pdep'' or ''dep''.');

end

if nargout>3,

%compute adjusted p-values; This can be a bit computationally intensive

adj_p=zeros(1,m)*NaN;

[wtd_p_sorted, wtd_p_sindex] = sort( wtd_p );

nextfill = 1;

for k = 1 : m

if wtd_p_sindex(k)>=nextfill

adj_p(nextfill:wtd_p_sindex(k)) = wtd_p_sorted(k);

nextfill = wtd_p_sindex(k)+1;

if nextfill>m

break;

end;

end;

end;

adj_p=reshape(adj_p(unsort_ids),s);

end

rej=p_sorted<=thresh;

max_id=find(rej,1,'last'); %find greatest significant pvalue

if isempty(max_id),

crit_p=0;

h=pvals*0;

adj_ci_cvrg=NaN;

else

crit_p=p_sorted(max_id);

h=pvals<=crit_p;

adj_ci_cvrg=1-thresh(max_id);

end

if strcmpi(report,'yes'),

n_sig=sum(p_sorted<=crit_p);

if n_sig==1,

fprintf('Out of %d tests, %d is significant using a false discovery rate of %f.\n',m,n_sig,q);

else

fprintf('Out of %d tests, %d are significant using a false discovery rate of %f.\n',m,n_sig,q);

end

if strcmpi(method,'pdep'),

fprintf('FDR/FCR procedure used is guaranteed valid for independent or positively dependent tests.\n');

else

fprintf('FDR/FCR procedure used is guaranteed valid for independent or dependent tests.\n');

end

end

Supplementary Materials – components in the tCCA latent space


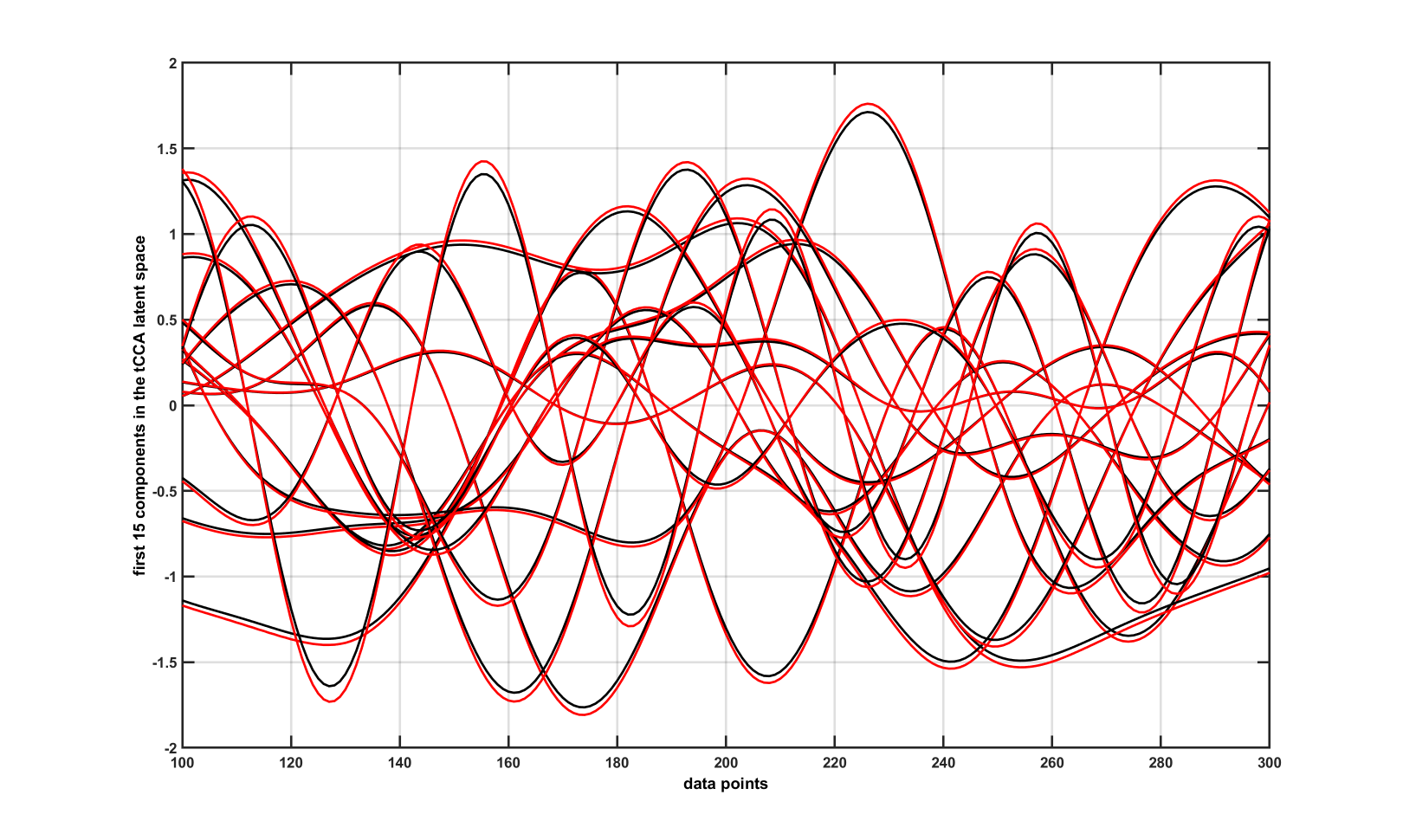


Figure S1: An illustrative plot of the 15 components or sources (greater than the correlation threshold, 0.99) in the tCCA latent space where red are the EEG bandpower (1-40Hz) sources and the black lines are the corresponding HbO sources. The 15 EEG sources were used as the regressors alongwith short-separation nuisance regressors in the GLM to reconstruct the HbO signal.

(A)

| **MS 1** | 0 | 0.217544 | **0.382456** | 0.098246 | 0.196491 | 0.105263 |
| --- | --- | --- | --- | --- | --- | --- |
| **MS 2** | 0.331579 | 0 | 0.252632 | 0.115789 | 0.152632 | 0.147368 |
| **MS 3** | **0.386029** | 0.220588 | 0 | 0.132353 | 0.191176 | 0.069853 |
| **MS 4** | 0.229358 | 0.220183 | 0.330275 | 0 | 0.137615 | 0.082569 |
| **MS 5** | 0.350282 | 0.124294 | 0.310734 | 0.079096 | 0 | 0.135593 |
| **MS 6** | 0.263636 | 0.209091 | 0.218182 | 0.081818 | 0.227273 | 0 |
|  | **MS 1** | **MS 2** | **MS 3** | **MS 4** | **MS 5** | **MS 6** |

(B)

| **MS 1** | 0 | 0.198113 | 0.349057 | 0.150943 | 0.207547 | 0.09434 |
| --- | --- | --- | --- | --- | --- | --- |
| **MS 2** | 0.228571 | 0 | **0.414286** | 0.114286 | 0.142857 | 0.1 |
| **MS 3** | 0.291045 | 0.186567 | 0 | 0.171642 | 0.30597 | 0.044776 |
| **MS 4** | 0.227273 | 0.136364 | 0.348485 | 0 | 0.257576 | 0.030303 |
| **MS 5** | 0.308511 | 0.106383 | **0.404255** | 0.138298 | 0 | 0.042553 |
| **MS 6** | 0.241379 | 0.172414 | 0.241379 | 0.206897 | 0.137931 | 0 |
|  | **MS 1** | **MS 2** | **MS 3** | **MS 4** | **MS 5** | **MS 6** |

(C)

| **MS 1** | 0 | 0.181818 | 0.272727 | 0.090909 | 0.363636 | 0.090909 |
| --- | --- | --- | --- | --- | --- | --- |
| **MS 2** | 0.333333 | 0 | 0.222222 | 0.222222 | 0.111111 | 0.111111 |
| **MS 3** | **0.444444** | 0.222222 | 0 | 0 | 0 | 0.333333 |
| **MS 4** | **0.666667** | 0.333333 | 0 | 0 | 0 | 0 |
| **MS 5** | 0.285714 | 0.142857 | 0.285714 | 0 | 0 | 0.285714 |
| **MS 6** | 0 | 0.428571 | 0.428571 | 0 | 0.142857 | 0 |
|  | **MS 1** | **MS 2** | **MS 3** | **MS 4** | **MS 5** | **MS 6** |

(D)

| **MS 1** | 0 | 0 | 0.6 | 0.4 | 0 | 0 |
| --- | --- | --- | --- | --- | --- | --- |
| **MS 2** | 0 | 0 | 0 | 0 | 0 | 0 |
| **MS 3** | 0.5 | 0 | 0 | 0.166667 | 0.333333 | 0 |
| **MS 4** | 0 | 0 | 0.666667 | 0 | 0 | 0.333333 |
| **MS 5** | 0 | 0 | **0.999999** | 0 | 0 | 0 |
| **MS 6** | **0.999999** | 0 | 0 | 0 | 0 | 0 |
|  | **MS 1** | **MS 2** | **MS 3** | **MS 4** | **MS 5** | **MS 6** |

Figure S2: Statistics on the transition probabilities between microstate (MS) classes at the group level, (A) during the 10-sec at the start of the FLS complex task in novices, (B) during the 10-sec at the start of the FLS complex task in experts, (C) during the 10-sec in the error epoch in novices, (D) during the 10-sec in the error epoch in experts. The rows denote the ‘from’ microstate and the columns denote the ‘to’ microstate.


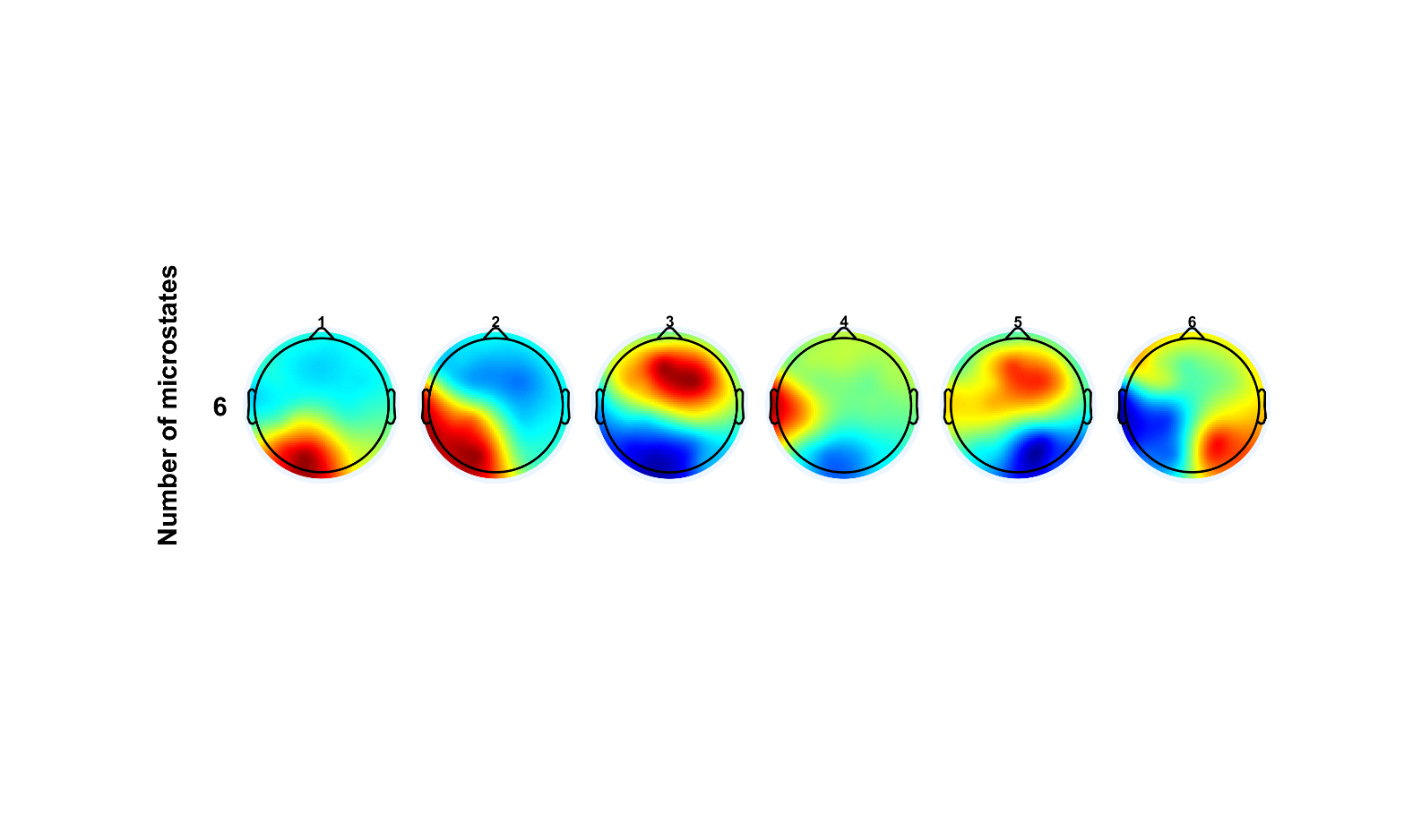


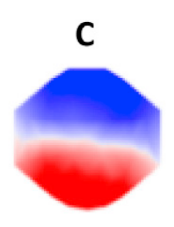

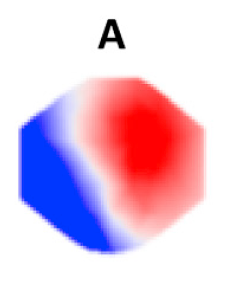

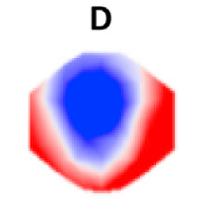
**
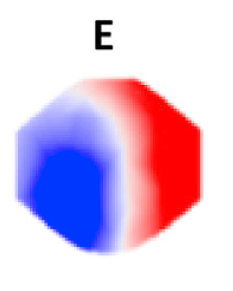
**
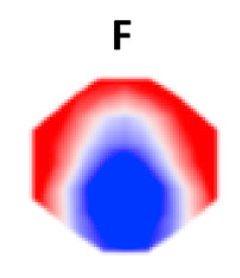

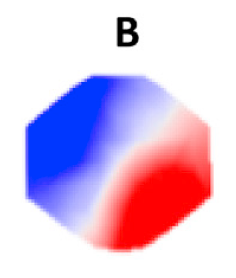


Figure S3: The six microstate prototypes shown in the top row and the topographically similar microstates from Brechet and colleagues (Bréchet et al., 2019) shown in the bottom row. In Brechet and colleagues (Bréchet et al., 2019), microstate A showed left-lateralized activity in the superior temporal gyrus (STG), the medial prefrontal cortex (MPFC) and the occipital gyri (OCG). Microstate B showed main activity in OCG and in the medial part of the parietal cortex. The sources of microstate C were located bilaterally in the lateral part of the parietal cortex including both the supramarginal gyrus (SMG) and angular gyrus (AG). The sources of microstate D showed main activity bilaterally in the inferior frontal gyrus (IFG), dorsal anterior cingulate cortex (dACC), and superior parietal lobule (SPL)/intraparietal sulcus (IPS). Strongest activity for microstate E was found in the right MPFC. Finally, microstate F showed bilateral activity in the MPFC.

Table S1: R - the correlation coefficient of the GLM fit to the data (#Channels x HbO) in HOMER3 for the novices, N01-N13

| **Source #** | **Detector #** | **N01** | **N02** | **N03** | **N04** | **N05** | **N06** | **N07** | **N08** | **N09** | **N10** | **N11** | **N12** | **N13** |
| --- | --- | --- | --- | --- | --- | --- | --- | --- | --- | --- | --- | --- | --- | --- |
| 1 | 1 | 0.35 | 0.35 | 0.35 | 0.27 | 0.28 | 0.29 | 0.26 | 0.25 | 0.37 | 0.34 | 0.24 | 0.45 | 0.42 |
| 1 | 15 | 0.32 | 0.35 | 0.33 | 0.31 | 0.29 | 0.33 | 0.31 | 0.30 | 0.32 | 0.36 | 0.28 | 0.25 | 0.41 |
| 1 | 16 | 0.34 | 0.34 | 0.30 | 0.28 | 0.33 | 0.34 | 0.33 | 0.32 | 0.39 | 0.32 | 0.35 | 0.33 | 0.30 |
| 2 | 2 | 0.19 | 0.20 | 0.29 | 0.31 | 0.34 | 0.33 | 0.25 | 0.27 | 0.32 | 0.29 | 0.23 | 0.39 | 0.28 |
| 2 | 3 | 0.34 | 0.33 | 0.35 | 0.40 | 0.31 | 0.32 | 0.40 | 0.26 | 0.32 | 0.30 | 0.33 | 0.30 | 0.39 |
| 2 | 17 | 0.30 | 0.29 | 0.24 | 0.34 | 0.27 | 0.29 | 0.35 | 0.29 | 0.29 | 0.31 | 0.33 | 0.27 | 0.39 |
| 3 | 1 | 0.34 | 0.24 | 0.38 | 0.30 | 0.47 | 0.33 | 0.45 | 0.35 | 0.28 | 0.32 | 0.28 | 0.41 | 0.53 |
| 3 | 3 | 0.31 | 0.26 | 0.27 | 0.36 | 0.38 | 0.29 | 0.32 | 0.29 | 0.37 | 0.32 | 0.24 | 0.37 | 0.43 |
| 3 | 5 | 0.34 | 0.29 | 0.30 | 0.26 | 0.32 | 0.34 | 0.39 | 0.22 | 0.64 | 0.22 | 0.33 | 0.27 | 0.33 |
| 4 | 1 | 0.28 | 0.41 | 0.22 | 0.31 | 0.21 | 0.33 | 0.34 | 0.21 | 0.36 | 0.28 | 0.23 | 0.32 | 0.36 |
| 4 | 3 | 0.22 | 0.26 | 0.22 | 0.30 | 0.27 | 0.30 | 0.25 | 0.25 | 0.31 | 0.36 | 0.27 | 0.30 | 0.36 |
| 4 | 15 | 0.31 | 0.30 | 0.25 | 0.37 | 0.21 | 0.30 | 0.30 | 0.33 | 0.37 | 0.36 | 0.31 | 0.26 | 0.36 |
| 5 | 2 | 0.29 | 0.36 | 0.28 | 0.41 | 0.28 | 0.33 | 0.20 | 0.27 | 0.37 | 0.28 | 0.32 | 0.39 | 0.39 |
| 5 | 3 | 0.37 | 0.27 | 0.50 | 0.32 | 0.28 | 0.32 | 0.37 | 0.35 | 0.39 | 0.31 | 0.30 | 0.51 | 0.45 |
| 5 | 4 | 0.33 | 0.28 | 0.29 | 0.30 | 0.25 | 0.27 | 0.32 | 0.21 | 0.41 | 0.32 | 0.26 | 0.38 | 0.38 |
| 5 | 5 | 0.43 | 0.36 | 0.25 | 0.27 | 0.34 | 0.29 | 0.33 | 0.23 | 0.53 | 0.30 | 0.32 | 0.45 | 0.33 |
| 6 | 4 | 0.29 | 0.23 | 0.27 | 0.28 | 0.24 | 0.21 | 0.28 | 0.28 | 0.36 | 0.31 | 0.28 | 0.43 | 0.38 |
| 6 | 5 | 0.37 | 0.48 | 0.27 | 0.31 | 0.44 | 0.27 | 0.29 | 0.25 | 0.50 | 0.29 | 0.36 | 0.45 | 0.37 |
| 6 | 6 | 0.29 | 0.35 | 0.24 | 0.37 | 0.39 | 0.19 | 0.30 | 0.29 | 0.40 | 0.22 | 0.25 | 0.29 | 0.38 |
| 6 | 18 | 0.41 | 0.26 | 0.22 | 0.28 | 0.20 | 0.28 | 0.31 | 0.27 | 0.39 | 0.27 | 0.31 | 0.35 | 0.29 |
| 7 | 2 | 0.40 | 0.24 | 0.34 | 0.33 | 0.21 | 0.31 | 0.33 | 0.25 | 0.41 | 0.31 | 0.31 | 0.36 | 0.37 |
| 7 | 4 | 0.34 | 0.25 | 0.27 | 0.30 | 0.38 | 0.40 | 0.30 | 0.31 | 0.47 | 0.33 | 0.25 | 0.29 | 0.47 |
| 7 | 7 | 0.35 | 0.28 | 0.27 | 0.30 | 0.32 | 0.36 | 0.34 | 0.29 | 0.50 | 0.33 | 0.26 | 0.39 | 0.37 |
| 8 | 4 | 0.28 | 0.27 | 0.19 | 0.26 | 0.28 | 0.38 | 0.26 | 0.24 | 0.43 | 0.37 | 0.28 | 0.33 | 0.48 |
| 8 | 6 | 0.25 | 0.31 | 0.25 | 0.27 | 0.22 | 0.31 | 0.35 | 0.25 | 0.38 | 0.29 | 0.28 | 0.35 | 0.50 |
| 8 | 7 | 0.33 | 0.22 | 0.26 | 0.25 | 0.29 | 0.31 | 0.33 | 0.26 | 0.29 | 0.39 | 0.34 | 0.41 | 0.43 |
| 8 | 19 | 0.30 | 0.31 | 0.27 | 0.31 | 0.25 | 0.32 | 0.26 | 0.20 | 0.37 | 0.34 | 0.29 | 0.33 | 0.43 |
| 9 | 8 | 0.30 | 0.28 | 0.39 | 0.31 | 0.29 | 0.31 | 0.30 | 0.30 | 0.48 | 0.37 | 0.23 | 0.24 | 0.40 |
| 9 | 15 | 0.27 | 0.35 | 0.35 | 0.32 | 0.25 | 0.27 | 0.36 | 0.25 | 0.32 | 0.24 | 0.27 | 0.29 | 0.36 |
| 9 | 20 | 0.27 | 0.28 | 0.25 | 0.35 | 0.26 | 0.27 | 0.53 | 0.32 | 0.40 | 0.31 | 0.29 | 0.45 | 0.33 |
| 10 | 8 | 0.32 | 0.35 | 0.37 | 0.33 | 0.27 | 0.31 | 0.25 | 0.27 | 0.32 | 0.30 | 0.30 | 0.35 | 0.35 |
| 10 | 9 | 0.24 | 0.30 | 0.24 | 0.31 | 0.34 | 0.28 | 0.32 | 0.32 | 0.30 | 0.18 | 0.20 | 0.35 | 0.51 |
| 10 | 13 | 0.33 | 0.24 | 0.31 | 0.30 | 0.23 | 0.29 | 0.43 | 0.28 | 0.35 | 0.29 | 0.30 | 0.49 | 0.36 |
| 10 | 21 | 0.35 | 0.20 | 0.19 | 0.29 | 0.26 | 0.40 | 0.28 | 0.38 | 0.27 | 0.33 | 0.32 | 0.35 | 0.54 |
| 11 | 9 | 0.31 | 0.23 | 0.30 | 0.41 | 0.28 | 0.36 | 0.27 | 0.23 | 0.35 | 0.32 | 0.27 | 0.36 | 0.42 |
| 11 | 10 | 0.30 | 0.35 | 0.45 | 0.33 | 0.29 | 0.24 | 0.28 | 0.23 | 0.46 | 0.36 | 0.27 | 0.33 | 0.27 |
| 12 | 8 | 0.40 | 0.31 | 0.33 | 0.25 | 0.24 | 0.31 | 0.33 | 0.30 | 0.47 | 0.26 | 0.29 | 0.24 | 0.37 |
| 12 | 9 | 0.32 | 0.28 | 0.34 | 0.29 | 0.27 | 0.33 | 0.19 | 0.37 | 0.39 | 0.24 | 0.26 | 0.33 | 0.42 |
| 12 | 15 | 0.32 | 0.33 | 0.46 | 0.37 | 0.24 | 0.27 | 0.32 | 0.28 | 0.38 | 0.27 | 0.33 | 0.31 | 0.34 |
| 13 | 9 | 0.41 | 0.24 | 0.33 | 0.33 | 0.32 | 0.36 | 0.28 | 0.27 | 0.32 | 0.31 | 0.23 | 0.38 | 0.47 |
| 13 | 10 | 0.43 | 0.20 | 0.27 | 0.39 | 0.32 | 0.37 | 0.21 | 0.26 | 0.30 | 0.25 | 0.33 | 0.36 | 0.33 |
| 13 | 12 | 0.25 | 0.23 | 0.28 | 0.29 | 0.35 | 0.40 | 0.27 | 0.22 | 0.41 | 0.24 | 0.30 | 0.37 | 0.46 |
| 13 | 13 | 0.37 | 0.40 | 0.49 | 0.32 | 0.25 | 0.36 | 0.32 | 0.29 | 0.34 | 0.21 | 0.32 | 0.32 | 0.33 |
| 13 | 22 | 0.27 | 0.20 | 0.26 | 0.29 | 0.30 | 0.34 | 0.33 | 0.20 | 0.31 | 0.34 | 0.32 | 0.28 | 0.32 |
| 14 | 12 | 0.33 | 0.22 | 0.33 | 0.36 | 0.35 | 0.27 | 0.37 | 0.30 | 0.46 | 0.22 | 0.32 | 0.29 | 0.36 |
| 14 | 13 | 0.37 | 0.53 | 0.37 | 0.33 | 0.22 | 0.28 | 0.26 | 0.26 | 0.35 | 0.30 | 0.34 | 0.48 | 0.22 |
| 14 | 14 | 0.31 | 0.20 | 0.32 | 0.33 | 0.30 | 0.26 | 0.34 | 0.20 | 0.40 | 0.34 | 0.29 | 0.30 | 0.41 |
| 15 | 10 | 0.32 | 0.21 | 0.25 | 0.31 | 0.34 | 0.21 | 0.27 | 0.26 | 0.43 | 0.35 | 0.28 | 0.32 | 0.33 |
| 15 | 11 | 0.32 | 0.26 | 0.39 | 0.32 | 0.35 | 0.30 | 0.29 | 0.33 | 0.44 | 0.32 | 0.35 | 0.42 | 0.59 |
| 15 | 12 | 0.32 | 0.28 | 0.24 | 0.31 | 0.32 | 0.38 | 0.42 | 0.23 | 0.34 | 0.30 | 0.18 | 0.37 | 0.34 |
| 16 | 11 | 0.35 | 0.24 | 0.30 | 0.28 | 0.30 | 0.31 | 0.35 | 0.22 | 0.41 | 0.33 | 0.28 | 0.43 | 0.37 |
| 16 | 12 | 0.30 | 0.18 | 0.30 | 0.30 | 0.31 | 0.29 | 0.33 | 0.19 | 0.46 | 0.24 | 0.28 | 0.32 | 0.38 |
| 16 | 14 | 0.28 | 0.24 | 0.26 | 0.31 | 0.33 | 0.26 | 0.40 | 0.25 | 0.44 | 0.21 | 0.32 | 0.34 | 0.34 |
| 16 | 23 | 0.26 | 0.28 | 0.28 | 0.32 | 0.32 | 0.23 | 0.23 | 0.29 | 0.24 | 0.44 | 0.34 | 0.31 | 0.33 |

Table S2: R - the correlation coefficient of the GLM fit to the data (#Channels x HbO) in HOMER3 for the experts, E01-E09

| **Source #** | **Detector #** | **E01** | **E02** | **E03** | **E04** | **E05** | **E06** | **E07** | **E08** | **E09** |
| --- | --- | --- | --- | --- | --- | --- | --- | --- | --- | --- |
| 1 | 1 | 0.49 | 0.53 | 0.51 | 0.51 | 0.51 | 0.49 | 0.51 | 0.51 | 0.49 |
| 1 | 15 | 0.43 | 0.60 | 0.57 | 0.53 | 0.49 | 0.52 | 0.38 | 0.38 | 0.44 |
| 1 | 16 | 0.37 | 0.47 | 0.50 | 0.38 | 0.48 | 0.40 | 0.44 | 0.44 | 0.34 |
| 2 | 2 | 0.45 | 0.48 | 0.47 | 0.50 | 0.50 | 0.49 | 0.47 | 0.47 | 0.43 |
| 2 | 3 | 0.49 | 0.36 | 0.45 | 0.47 | 0.49 | 0.45 | 0.41 | 0.41 | 0.53 |
| 2 | 17 | 0.27 | 0.42 | 0.31 | 0.51 | 0.49 | 0.57 | 0.38 | 0.38 | 0.42 |
| 3 | 1 | 0.32 | 0.38 | 0.37 | 0.50 | 0.45 | 0.55 | 0.31 | 0.31 | 0.45 |
| 3 | 3 | 0.44 | 0.35 | 0.40 | 0.39 | 0.46 | 0.66 | 0.54 | 0.54 | 0.48 |
| 3 | 5 | 0.38 | 0.46 | 0.59 | 0.55 | 0.28 | 0.36 | 0.51 | 0.51 | 0.48 |
| 4 | 1 | 0.43 | 0.49 | 0.43 | 0.52 | 0.45 | 0.60 | 0.48 | 0.48 | 0.46 |
| 4 | 3 | 0.36 | 0.52 | 0.42 | 0.48 | 0.47 | 0.55 | 0.34 | 0.34 | 0.46 |
| 4 | 15 | 0.46 | 0.54 | 0.56 | 0.47 | 0.48 | 0.51 | 0.40 | 0.40 | 0.46 |
| 5 | 2 | 0.54 | 0.49 | 0.36 | 0.46 | 0.47 | 0.47 | 0.48 | 0.48 | 0.49 |
| 5 | 3 | 0.35 | 0.39 | 0.41 | 0.49 | 0.51 | 0.58 | 0.52 | 0.52 | 0.50 |
| 5 | 4 | 0.47 | 0.42 | 0.41 | 0.48 | 0.46 | 0.55 | 0.49 | 0.49 | 0.45 |
| 5 | 5 | 0.48 | 0.47 | 0.43 | 0.47 | 0.42 | 0.54 | 0.47 | 0.47 | 0.39 |
| 6 | 4 | 0.50 | 0.52 | 0.38 | 0.43 | 0.45 | 0.59 | 0.39 | 0.39 | 0.37 |
| 6 | 5 | 0.47 | 0.44 | 0.46 | 0.37 | 0.32 | 0.46 | 0.45 | 0.45 | 0.43 |
| 6 | 6 | 0.42 | 0.44 | 0.41 | 0.62 | 0.49 | 0.58 | 0.46 | 0.46 | 0.47 |
| 6 | 18 | 0.34 | 0.30 | 0.34 | 0.39 | 0.41 | 0.37 | 0.34 | 0.34 | 0.54 |
| 7 | 2 | 0.53 | 0.40 | 0.38 | 0.32 | 0.50 | 0.46 | 0.47 | 0.47 | 0.51 |
| 7 | 4 | 0.50 | 0.39 | 0.44 | 0.55 | 0.53 | 0.56 | 0.38 | 0.38 | 0.46 |
| 7 | 7 | 0.52 | 0.44 | 0.39 | 0.50 | 0.48 | 0.62 | 0.42 | 0.42 | 0.49 |
| 8 | 4 | 0.45 | 0.39 | 0.40 | 0.58 | 0.53 | 0.55 | 0.39 | 0.39 | 0.47 |
| 8 | 6 | 0.40 | 0.42 | 0.51 | 0.51 | 0.57 | 0.59 | 0.41 | 0.41 | 0.45 |
| 8 | 7 | 0.49 | 0.39 | 0.36 | 0.49 | 0.47 | 0.50 | 0.43 | 0.43 | 0.49 |
| 8 | 19 | 0.44 | 0.38 | 0.37 | 0.37 | 0.42 | 0.35 | 0.36 | 0.36 | 0.38 |
| 9 | 8 | 0.36 | 0.49 | 0.35 | 0.50 | 0.48 | 0.37 | 0.37 | 0.37 | 0.52 |
| 9 | 15 | 0.47 | 0.51 | 0.46 | 0.39 | 0.46 | 0.47 | 0.36 | 0.36 | 0.41 |
| 9 | 20 | 0.42 | 0.52 | 0.43 | 0.48 | 0.48 | 0.55 | 0.35 | 0.35 | 0.51 |
| 10 | 8 | 0.43 | 0.44 | 0.43 | 0.51 | 0.50 | 0.56 | 0.44 | 0.44 | 0.41 |
| 10 | 9 | 0.35 | 0.34 | 0.38 | 0.44 | 0.51 | 0.51 | 0.49 | 0.49 | 0.46 |
| 10 | 13 | 0.38 | 0.48 | 0.57 | 0.61 | 0.38 | 0.45 | 0.45 | 0.45 | 0.35 |
| 10 | 21 | 0.40 | 0.37 | 0.52 | 0.43 | 0.43 | 0.60 | 0.44 | 0.44 | 0.36 |
| 11 | 9 | 0.42 | 0.33 | 0.33 | 0.39 | 0.53 | 0.53 | 0.44 | 0.44 | 0.40 |
| 11 | 10 | 0.46 | 0.50 | 0.51 | 0.59 | 0.49 | 0.53 | 0.51 | 0.51 | 0.45 |
| 12 | 8 | 0.43 | 0.45 | 0.48 | 0.53 | 0.45 | 0.46 | 0.46 | 0.46 | 0.50 |
| 12 | 9 | 0.49 | 0.51 | 0.47 | 0.43 | 0.44 | 0.72 | 0.56 | 0.56 | 0.45 |
| 12 | 15 | 0.53 | 0.52 | 0.48 | 0.53 | 0.46 | 0.52 | 0.36 | 0.36 | 0.35 |
| 13 | 9 | 0.47 | 0.41 | 0.45 | 0.35 | 0.42 | 0.56 | 0.51 | 0.51 | 0.37 |
| 13 | 10 | 0.47 | 0.46 | 0.37 | 0.43 | 0.49 | 0.57 | 0.40 | 0.40 | 0.44 |
| 13 | 12 | 0.51 | 0.52 | 0.39 | 0.57 | 0.49 | 0.52 | 0.38 | 0.38 | 0.40 |
| 13 | 13 | 0.51 | 0.37 | 0.51 | 0.53 | 0.49 | 0.41 | 0.35 | 0.35 | 0.34 |
| 13 | 22 | 0.36 | 0.40 | 0.43 | 0.31 | 0.50 | 0.41 | 0.40 | 0.40 | 0.38 |
| 14 | 12 | 0.53 | 0.53 | 0.47 | 0.62 | 0.48 | 0.59 | 0.33 | 0.33 | 0.45 |
| 14 | 13 | 0.39 | 0.43 | 0.63 | 0.45 | 0.45 | 0.47 | 0.50 | 0.50 | 0.30 |
| 14 | 14 | 0.61 | 0.40 | 0.47 | 0.62 | 0.52 | 0.55 | 0.38 | 0.38 | 0.37 |
| 15 | 10 | 0.50 | 0.50 | 0.47 | 0.55 | 0.54 | 0.51 | 0.38 | 0.38 | 0.49 |
| 15 | 11 | 0.56 | 0.34 | 0.38 | 0.42 | 0.54 | 0.49 | 0.45 | 0.45 | 0.48 |
| 15 | 12 | 0.58 | 0.42 | 0.42 | 0.47 | 0.53 | 0.55 | 0.38 | 0.38 | 0.46 |
| 16 | 11 | 0.52 | 0.44 | 0.35 | 0.42 | 0.53 | 0.49 | 0.46 | 0.46 | 0.39 |
| 16 | 12 | 0.52 | 0.44 | 0.44 | 0.58 | 0.44 | 0.59 | 0.39 | 0.39 | 0.42 |
| 16 | 14 | 0.51 | 0.41 | 0.37 | 0.59 | 0.44 | 0.58 | 0.49 | 0.49 | 0.48 |
| 16 | 23 | 0.36 | 0.42 | 0.37 | 0.35 | 0.47 | 0.40 | 0.44 | 0.44 | 0.36 |
